# Low-density lipoprotein receptor-related protein 6 regulates cardiomyocyte-derived paracrine signaling to ameliorate cardiac fibrosis

**DOI:** 10.7150/thno.48787

**Published:** 2021-01-01

**Authors:** Xiang Wang, Yan Zou, Zhidan Chen, Yang Li, Le Pan, Ying Wang, Ming Liu, Chao Yin, Jian Wu, Chunjie Yang, Lei Zhang, Chenze Li, Zheyong Huang, Daowen Wang, Juying Qian, Junbo Ge, Yunzeng Zou, Hui Gong

**Affiliations:** 1 Shanghai Institute of Cardiovascular Diseases, Zhongshan Hospital, and Institutes of Biomedical Sciences, Fudan University, Shanghai 200032, China.; 2 Division of Cardiology, Department of Internal Medicine and Hubei Key Laboratory of Genetics and Molecular Mechanisms of Cardiological Disorders, Tongji Hospital, Tongji Medical College, Huazhong University of Science and Technology, Wuhan 430030, China.

**Keywords:** Pressure overload, Cardiac fibrosis, LRP6, Wnt5a, Wnt11, CTSD

## Abstract

**Rationale:** Maladaptive cardiac remodeling is a critical step in the progression of heart failure. Low-density lipoprotein receptor-related protein 6 (LRP6), a co-receptor of Wnt, has been implicated in cardiac protection. We aimed to study the role of cardiomyocyte-expressed LRP6 in cardiac remodeling under chronic pressure overload.

**Methods:** Cardiac parameters were analyzed in inducible cardiac-specific LRP6 overexpressing and control mice subjected to transverse aortic constriction (TAC).

**Results:** Cardiac LRP6 was increased at an early phase after TAC. Cardiomyocyte-specific LRP6 overexpression improved cardiac function and inhibited cardiac hypertrophy and fibrosis four weeks after TAC. The overexpression significantly inhibited β-catenin activation, likely contributing to the inhibitory effect on cardiac hypertrophy after TAC. LRP6 overexpression reduced the expression and secretion of Wnt5a and Wnt11 by cardiomyocytes, and knockdown of Wnt5a and Wnt11 greatly inhibited cardiac fibrosis and dysfunction under pressure overload *in vitro* and *in vivo*. Cardiomyocyte-expressed LRP6 interacted with cathepsin D (CTSD, a protease) and promoted the degradation of Wnt5a and Wnt11, inhibiting cardiac fibrosis and dysfunction induced by TAC. The protease inhibitor leupeptin attenuated the interaction between LRP6 and CTSD, enhanced the expression of Wnt5a and Wnt11, and deteriorated cardiac function and fibrosis in cardiomyocyte-specific LRP6-overexpressing mice under pressure overload. Mutants from human patients, P1427Q of LRP6 and G316R of CTSD significantly inhibited the interaction between LRP6 and CTSD and increased Wnt5a and Wnt11 expression.

**Conclusion:** Cardiomyocyte-expressed LRP6 promoted the degradation of Wnt5a and Wnt11 by regulating CTSD and inhibited cardiac fibrosis under pressure overload. Our study demonstrated a novel role of LRP6 as an anti-fibrosis regulator.

## Introduction

Maladaptive cardiac remodeling is a critical step in the progression of heart failure, and is driven by a constellation of cellular responses, including cardiomyocyte hypertrophy, inflammation, and fibrosis 1. Many signaling pathways are implicated in adverse cardiac remodeling at the cellular level in response to mechanical stress or neurohumoral factors associated with hypertension or ischemia 1-2. However, the molecular mechanisms underlying the transition from adaptive cardiac remodeling to dilated cardiomyopathy or heart failure remain largely unknown.

LRP6 is one of the Wnt co-receptors. In canonical Wnt/β-catenin signaling, Wnts bind to the heterodimeric receptor complex, consisting of Frizzled (FZD) and LRP5/6 proteins, preventing the degradation of β-catenin and allowing it to translocate to the nucleus and activate Wnt/β-catenin-targeted gene transcription 3. The canonical Wnt signaling has been implicated in regulating multiple cellular processes involved in metabolism, immune response, and the development of several cancers 4-8. We recently found that LRP6 was decreased in heart tissues of dilated cardiomyopathy (DCM) patients, and cardiac-specific deficiency of LRP6 induced cardiac dysfunction by inhibiting autophagy and fatty acid metabolism 9. Deficiency of LRP6 in cardiomyocytes predisposes the heart to lethal arrhythmia due to the degradation of connexin 43 independent of Wnt signaling 10, 11, suggesting that LRP6 has more diverse roles than other Wnt receptors. The deletion of LRP5/6 enhances cardiac ischemic injury, including apoptosis 12. Multiple functional mutations within the LRP6 gene have been identified to be linked to early coronary artery diseases (CAD) with hypercholesterolemia 13, indicating that LRP6 might play a critical role in myocardial infarction pathogenesis. Based on the evidence showing a significant role of LRP6 in cardiomyocytes, we hypothesized that LRP6 expressed in cardiomyocytes may protect the heart from maladaptive cardiac remodeling or cardiac dysfunction.

In this study, we generated an inducible cardiac-specific LRP6-overexpressing mouse model with pressure overload. We examined the effects of LRP6 on cardiac function and pathological remodeling under pressure overload and explored the potential underlying molecular mechanisms.

## Materials and Methods

### Inducible cardiac LRP6-overexpressing mice

Here we established transgenic mouse lines for Cre-inducible LRP6 expression. The transgenic construct consisted of CAG promoter followed by a lox P-flanked cassette of the chloramphenicol acetyltransferase (CAT) resistance fusion gene and a triple poly A transcription termination signal (STOP) 15. The full-length human LRP6 coding sequence was isolated from LRP6-pCS2-VSVG (Addgene 27282#) and inserted into the downstream of this cassette to construct CAG-CAT-LRP6 which was then directly sequenced to verify successful insertion of the full-length human LRP6 coding sequence. After purifying and linearizing, the construct was introduced by the pronuclear microinjection of fertilized blastocysts of FVBN mice. Four independent transgenic founder lines were validated by genotyping. We named these mice as LRP6^CAG^. The presence of the “STOP” signal blocked the expression of the downstream LRP6 gene in LRP6^CAG^. The mice were bred with α-myosin heavy chain (α-MHC) Mer-Cre-Mer Tg mice (α-MHC-MCM, termed MCM) were crossed to generate LRP6^CAG^/MCM mice. We generated four lines of LRP6^CAG^ mice (L1-L4-LRP6^CAG^). After α-MHC-MCM-mediated recombination and tamoxifen injection, the CAG promoter drove a high level of expression of LRP6 in cardiomyocytes (CMs). In the present study, tamoxifen (30 mg/kg/day) was injected into 6-8-week-old male L1-L4-LRP6^CAG^/MCM mice for three consecutive days, following which LRP6 overexpression was confirmed. In this study, L1-LRP6^CAG^/MCM mice injected with tamoxifen were termed LRP6 Over mice for analysis. The basal phenotype of MCM, LRP6^CAG^, and LRP6^CAG^/MCM mice after injection with tamoxifen was analyzed.

### Transverse aortic constriction (TAC) in mice

The pressure overload mouse model was built by TAC. To exclude the influence of estrogen, we chose male mice to perform TAC in the present study. Adult male mice, aged 8-10 weeks, were anesthetized with isoflurane, artificially ventilated, and then the aortic arch was separated and ligated by 6-0 nylon suture with a blunted 27-gauge needle to ensure the degree of narrowing. The needle was later pulled out. The sham group was subjected to the same surgical procedure except ligating the transverse aorta. Further analyses were performed at different time points after TAC. Echocardiography analysis was performed on all mice prior to sham or TAC operation.

### Echocardiography and hemodynamic analysis

The mice were anesthetized with isoflurane, and M-mode images were obtained with a VisualSonics Vevo2100 animal imaging instrument (VisualSonics, Inc.). Fractional shortening (FS) and ejection fraction (EF) were assessed. Heart rate (HR) was maintained at >400 bpm. The micro-nanocatheter was inserted into the right common carotid artery and finally inserted into the left ventricle (LV). A 1.4 F cardiac catheter (Millar Instruments, Inc.) was connected to a Power Laboratory system (AD Instruments, Castle Hill, Australia). Hemodynamic parameters, such as left ventricular end diastolic or systolic pressure and positive or negative dp/dt_max_ were measured and analyzed.

### Nano-HPLC-MS/MS analysis and database searching

The supernatant was transferred into a new tube and stored at -80 °C. Conditioned media (2 ml each) were concentrated into 100 μL using 3 kD MWCO spin column and 30 μL was mixed with 4× Laemmli buffer, and proteins were separated by 4-12% Bis-Tris gel at 130 V for 1.5 h. The gels were silver stained; the darker bands were observed in stretched LRP6-overexpressed CMs, and compared with the control group. The chosen bands were cut and digested overnight in the digestion solution (trypsin 12.5 ng/μL and 20 mM NH4HCO3). The digested peptides were lyophilized and re-suspended with 32μl solvent C (C: water with 0.1% formic acid; D: ACN with 0.1% formic acid), separated by nanoLC and analyzed by online electrospray tandem mass spectrometry. The experiment was performed on a Nano Aquity UPLC system (Waters Corporation, Milford, MA), which was connected to a quadrupole-Orbitrap mass spectrometer (Q-Exactive) (Thermo Fisher Scientific, Bremen, Germany) equipped with an online nano-electrospray ion source. The peptide sample (4 μl) was loaded onto the trap column (Thermo Scientific Acclaim PepMap C18, 100 μm × 2 cm) and subsequently separated on the analytical column (Acclaim PepMap C18, 75 μm × 25 cm) with a linear gradient from 5% D to 30% D in 85 min. The column was re-equilibrated at initial conditions for 5 min. The column flow rate was maintained at 300 nL/min, and the column temperature was maintained at 45 °C. The electrospray voltage of 2.0 kV versus the inlet of the mass spectrometer was used.

All MS/MS samples were analyzed using Mascot (Matrix Science, London, UK; version 2.3) set up to search the NCBI Rattus norvegicus database assuming the digestion enzyme trypsin. Mascot was searched with a fragment ion mass tolerance of 0.050 Da and a parent ion tolerance of 10.0 ppm. Carbamidomethyl of cysteine was specified in Mascot as fixed modifications. Oxidation of methionine was specified in Mascot as a variable modification.

### Mechanical stretch

The stretch device was used as described in our previous study 16. The silicone sheet (20×40 mm) was sterilized and coated with rat tail collagen in 0.1% acetic acid using an airbrush. CMs were cultured on these sheets for 2-3 days, and then deprived of serum for 24 hours. For stretching, the silicone sheet was fixed in the stretching frame (in a 150 mm culture dish) and stretched to 120% by uniaxial strain provided by stretching the sheet in the frame. The control groups were also grown on the silicone sheet without being subjected to stretch. The cells or the culture medium were harvested for analysis at specific time-points.

### Real-time PCR (RT-PCR) analysis

The mRNA expression level of genes was measured using RT-PCR. Total RNA was extracted from cardiac fibroblasts (CFs) or heart tissues using TRIzol reagent (Invitrogen, Carlsbad, CA). We used Nano2000 to determine RNA concentration and quality to ensure A260/280 was between 1.8 and 2.2, and 1 μg total RNA was used to obtain cDNA for RT-PCR by using the Toyobo RT-PCR kit. For relative quantitation of RNA, the SYBR Premix ExTaq kit (Cat#: RR420A, Takara, Japan) was used for RT-PCR. The primers were synthesized by Sangon Biotech (Shanghai, China) (Table S1). The specific PCR conditions were denaturation at 95 °C for 30 s, annealing at 55 °C for 40 s, and extension at 72 ° C for 60 s for 40 cycles. We used GAPDH as an internal control.

### Immunoprecipitation and Western blotting

Total protein was extracted from mouse heart tissues with the radioimmunoprecipitation assay (RIPA) buffer, quantitated using the BCA working reagent, and incubated overnight at 4 °C with 1 ug primary antibodies against LRP6, CTSD, or isotype immunoglobulin G (Cell Signaling Technology, Inc., 3900) as a control, with shaking. On the second day, 40 mL protein A/G sepharose beads (Sea Biotech, P001-2) were added, and the mixture was incubated for 2-3 h at 4 °C with rotation, followed by 1000 g centrifugation to collect protein mixture. The precipitates were washed 3-5 times with PBS, and the samples were analyzed by Western blotting. The target proteins were separated by SDS/PAGE and transferred to PVDF membranes. After blocking with 5% bovine serum albumin (BSA) for 1-2 h, the membranes were incubated overnight at 4 °C with primary antibodies (Table S2) followed by incubation with horseradish peroxidase-conjugated secondary antibodies (1:5000) for 1-2 h at room temperature. Following interaction, the pro-light chemiluminescent detection kit (Tiangen Biotech Inc., Beijing, China), was used to analyze the proteins with the LAS-3000 imaging system (FUJIFILM Inc., Tokyo, Japan).

### Histology

Cardiac hypertrophy and fibrosis were evaluated by Hematoxylin and Eosin (HE) and Masson's trichrome staining. The heart tissues were cut into 2 mm sections and fixed in 4% polyformaldehyde for at least 72 h at room temperature. After a series of dehydration and paraffin soaking steps, the heart tissues were embedded in paraffin, cut into 5-μm sections by Leica paraffin slicer, and subjected to HE and Masson's trichrome staining. The images were obtained by using the ImagePro Plus5.0 (Media Cybernetics, Rockville, MD) image analysis system to quantify the fibrotic area (blue area). Vascular fibrosis was calculated as a percentage of the entire tissue area = blue area in the vascular area to the entire tissue area; interstitial fibrosis was assessed as a percentage of the entire tissue area = blue area (excluding the vascular area) to the entire tissue area. Six images per left ventricle were obtained, and the images from 3-6 mice in each group were used for quantitative analysis.

### Isolation of cardiomyocytes and non-cardiomyocytes from adult mouse

We isolated adult mouse CMs according to the previously described procedure 14. MCM and LRP6 Over male mice aged about 8-10 weeks were anesthetized, and the thoracic cavity was opened to expose the heart. The inferior vena and cava-descending aorta were cut and then the heart was immediately flushed by injecting 7-10 mL of EDTA buffer. The heart was immediately digested by sequentially injecting 10 mL of EDTA buffer, 3ml of perfusion buffer, and 50 mL of collagenase buffer into the left ventricle from the aorta. After digestion, the heart tissue was cut into pieces of about 1mm^3^ and manually dissociated into single cardiomyocyte in 10 % BSA perfusion buffer. The cell suspension was filtered through a 100 μm cell strainer, and the CMs were collected by natural sedimentation for 20 min, and part of the supernatant was centrifuged at low speed to collect non-CMs.

### Cathepsin D (CTSD) activity analysis

We harvested the amount of tissue necessary for each assay and washed it in cold PBS. Subsequently, we re-suspended the tissue in 200-1000 uL of CTSD Cell Lysis Buffer and homogenized with a Dounce homogenizer on ice with 10-15 passes. The samples were centrifuged for 2- 5 min at 4 °C and maximum speed using a cold micro-centrifuge to remove any insoluble material. The clear tissue lysate was transferred into the reaction mix solution (reaction buffer 50 µL, substrate 2 µL and treated sample 48 µL) and incubated at 37 °C for 1 -2 h in the dark. Finally, CTSD activity was analyzed on a microplate reader (Ex/Em = 328/460 nm).

### Neonatal cardiomyocyte and cardiac fibroblast culture

We obtained CFs and primary CMs from the hearts of 1-3-day-old Sprague-Dawley (SD) rats by trypsin digestion. After washing with ice-cold PBS, the heart was cut into 1 mm^3^ pieces and digested with 0.125% trypsin. The digestion was repeated 6-7 times, and the cells were thoroughly mixed and plated in a sterile 10 cm culture dish. After 2 h of cell attachment, the attached CFs and CMs from the medium were collected. The adherent CFs or CMs were cultured in DMEM/F12 containing 10% FBS and 1% antibiotic for 24 h, and then the medium was changed every 48 h.

### siRNA transfection

We used siRNA to knockdown the expression of CTSD or Wnt5a and Wnt11 in CMs *in vitro*. The siRNAs targeted to rat CTSD (numbers 164, 464, 1248) were designed and synthesized by GenePharma (Shanghai, China). siPORT™ NeoFX™ Transfection Agent (Ambion Inc, Texas, U.S.A.) was used for the transfection of CTSD siRNA according to the manufacturer's protocols. Briefly, the CTSD siRNA (100 nM in 100 μL opti-DMEM) and transfection agent (5 in 100 mL opti-DMEM) were mixed and kept for 10 min at room temperature before adding the mixtures (200 μl) to cultured CMs. We designed three sequences to knockdown of CTSD in CMs and finally selected the si-CTSD (1248), sense: 5'-CCUGGGCGAUGUCUUUAUUTT-3'; antisense: 5'-AAUAAAGACAUCGCCCAGGTT-3'. siWnt5a, sense: 5'-CCACGCCAAGGGCUCCUAUTT-3', anti-sense: 5'-AUAGGAGCCCUUGGCGUGGTT-3'; siWnt11, sense 5'-CCAUCAGUCACACCAUTT-3', antisense: 5'-AUGGUGUGACUGAUGGUGGTT-3'.

### Adenovirus construction and transduction

To generate LRP6 adenovirus particles, the LRP6 sequence from LRP6 PCS2 (Addgene 27282#) was cloned into an adenovirus vector; the empty vector was used as the control. Adenoviral vectors with or without the LRP6 expression cassette were generated with the AdEasy XL Adenoviral Vector System (Hanheng Biotechnology Co., Ltd, Shanghai). The virus particles were packaged and amplified in HEK 293 cells. Our preliminary experiments showed that an MOI value of 30 had significant overexpression and did not affect myocardial cell status. LRP6 was overexpressed in CMs 48 h of transfection.

### Adeno-associated virus 9 vector (AAV9) construction

The siRNA-sequences targeted to mouse Wnt5a and Wnt11 were sub-cloned into AAV9 plasmid for constructing AAV9-shWnt5a/11 plasmids to generate AAV9 particles by Hanheng Biotechnology Co., Ltd (Shanghai, China). Sh-Scramble was used as a control. ShWnt5a sequence: CCGGGCTAATTCTTGGTGGTCTCTACTCGAGTAGAGACCACCAAGAATTAGCTTTTTG; shWnt11 sequence: CCGGCCTGTGAAGGACTCAGAACTTCTCGAGAAGTTCTGAGTCCTTCACAGGTTTTTG. The AAV9 viruses 8.8 × 10^11^ particles were injected into the tail vein of 6 week-old mice. After 2-3 weeks, a sham or TAC operation was performed.

### Mutation of LRP6 or CTSD generation and transfection

Human wild type LRP6 was obtained from Addgene (LRP6-pCS2-VSVG, 27282#). The LRP6 sequence was constructed into plasmid pcDNA3.1 modified with a 3*flag tag at the C terminus. We purchased plasmid pcDNA3.1 containing human CTSD modified with His from Synbio Technology (Suzhou, China). LRP6 mutants were generated by primer-mediated PCR mutagenesis and verified by sequence analysis. CTSD mutants were generated by the same method. The WT sequence and the presence of mutations were identified by sequence analysis. Human embryonic kidney (HEK) 293 T cells were cultured in 10cm plates. Twenty-four hours later, LRP6 or CTSD WT and mutant plasmids as well as the MESD plasmid were transfected into the cells in indicated combinations. MESD facilitates membrane localization of LRP6. In each transfection experiment, the total DNA concentration was kept constant. After 24 h, cells were lysed and immunoprecipitation and Western blotting were performed. Under stretch, LRP6 or CTSD WT and mutant plasmids as well as MESD plasmid were transfected into the COS7 cells plated on silicone sheets. Typically, each experiment was repeated in at least three different batches of cultures.

### Statistics

We used histograms to test for normality in the present study. For expression levels *in vivo* and *in vitro*, all graphs were approximately bell-shaped and symmetric about the mean. GraphPad Prism 6.0 was used for statistical analysis. Data were expressed as mean ± standard errors (SEM). For the values in cardiac-specific LRP6 overexpressing mice and MCM mice with sham or TAC operation, two-way ANOVA with repeated measures was performed for a comparison between groups. For comparisons of 2 groups, the unpaired Student's *t-*test was used when appropriate. For comparisons among 3 or more groups, we performed one-way ANOVA followed by the Tukey post-test. Statistical significance was considered as *p* < 0.05.

### Study approval

All animal experiment protocols were approved by the Animal Care and Use Committee of Zhongshan Hospital, Fudan University. The mutants LRP6 (P1104R) and CTSD (G316R) were detected in DCM patients in Tongji Hospital affiliated with Tongji Medical School (Wuhan, China). The study was registered on the clinical trial website and approved by the ethics committee of Huazhong University of Science and Technology (Approval No: TJ-C20181001). All patients or their families provided written informed consent.

## Results

### Cardiomyocyte-specific LRP6 overexpression prevents cardiac dysfunction induced by pressure overload

**Figure 1:**
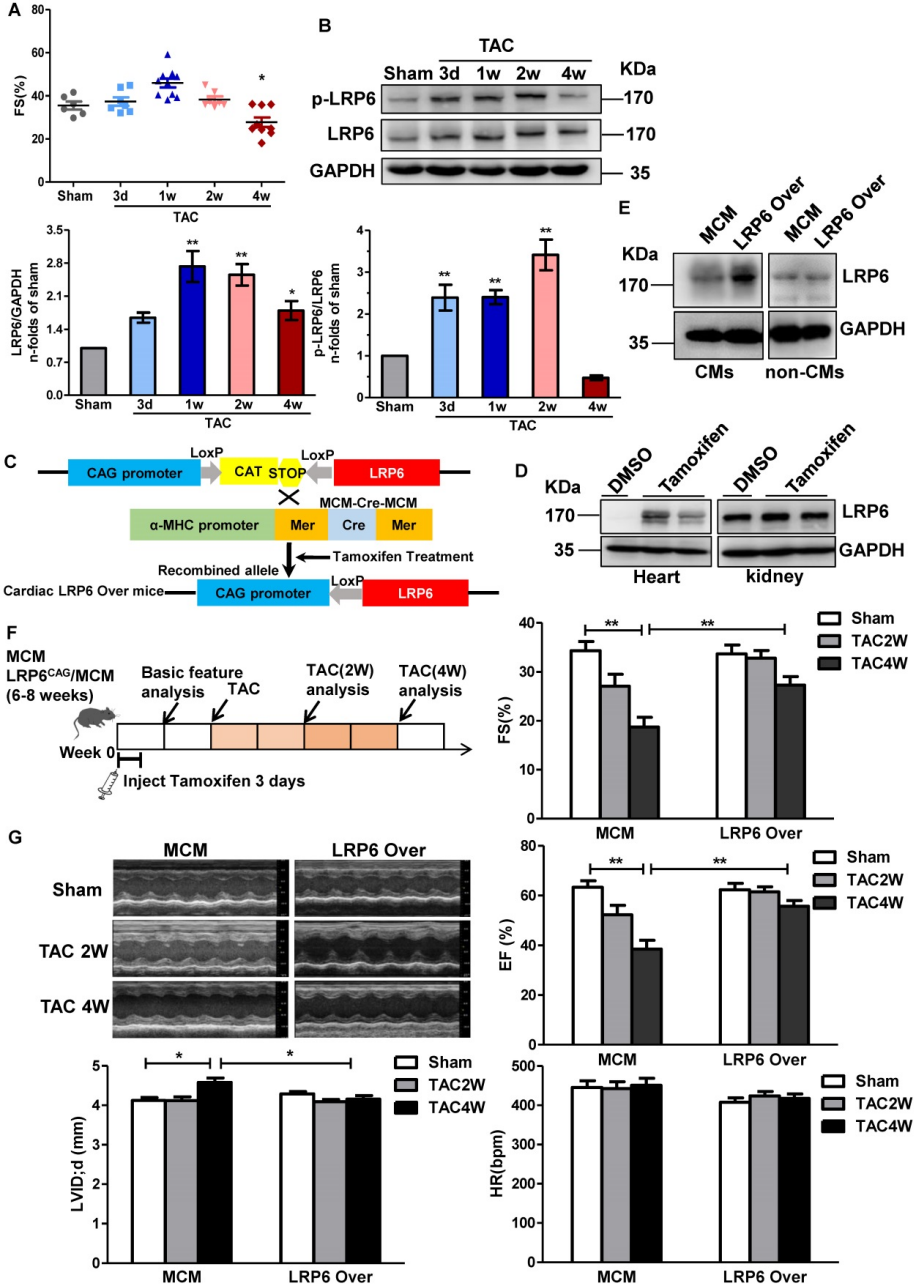
** Cardiomyocyte-specific overexpression of LRP6 prevents cardiac dysfunction induced by pressure overload.** (**A**), Echocardiographic analysis of fraction shortening (FS) in mice at different time-points (3d, 1w, 2w, and 4w) after TAC. *p < 0.05 *vs* Sham group; n = 6-10 mice/each group. (**B**), Western blot analysis of p-LRP6 and LRP6 levels in left ventricular tissues from mice at different time-points (3d, 1w, 2w, and 4w) after TAC. *p < 0.05; **p < 0.01 vs sham group; n = 4 mice/ each group. (**C**), Summary of the generation of tamoxifen-inducible cardiac-specific LRP6 overexpressing mice. (**D**), Representative images of Western blot analysis of LRP6 expression in heart and kidney tissues from LRP6^CAG^/MCM after injection of tamoxifen or DMSO for 3 consecutive days. (**E**), Representative images of Western blot analysis of LRP6 expression in isolated CMs and non-CMs from tamoxifen-injected LRP6^CAG^/MCM (LRP6 Over) or -MCM (MCM) mice. CMs: cardiomyocytes. (**F**), Overall strategy of exploring the effects of cardiac LRP6 overexpression in TAC mice. (**G**), Echocardiographic analysis of cardiac function in tamoxifen-injected LRP6^CAG^/MCM (LRP6 Over) or -MCM (MCM) mice at 2 weeks and 4 weeks after TAC or sham operation. FS: fraction shortening; EF: ejection fraction; LVID;d: left ventricular diastolic internal diameter. HR: heart rate; *p < 0.05; **p < 0.01; n = 7-12/each group.

To explore the expression profile of cardiac LRP6 during the pressure overload, we examined cardiac LRP6 expression at day 3, 7, 14, and 28 after TAC compared with the sham operation. Fraction shortening (FS) and ejection fraction (EF) were maintained from day 3 to 2 weeks, but declined at 4 weeks after TAC (Figure 1A, S1A), indicating that pressure overload induced cardiac dysfunction at 4 weeks after TAC. Cardiac LRP6 expression was increased from 1 week, compared with the sham operation, while p-LRP6 level was increased from day 3, reached a plateau at 2 weeks after TAC (Figure 1B). We also analyzed the expression of active-β-catenin (canonical Wnt signaling), Col1, matrix metalloproteinase-2 (MMP2), and MMP9 following TAC. MMP-9 is usually increased under conditions that trigger tissue remodeling, including cardiac remodeling 17. These proteins were increased from 1 week and maintained higher levels from 2 weeks to 4 weeks after TAC than in the sham group. The total β-catenin expression did not show an obvious alteration from 3 days to 4 weeks after TAC (Figure S1B). RT-PCR results indicated that Col1 and MMP2 mRNA were increased from 3 days to 4 weeks, and Col 3 and MMP9 mRNAs were increased at 2 weeks and 4 weeks following TAC (Figure S1C). The data suggest that cardiomyocyte-expressed LRP6 may play a critical role in adaptive response during pressure overload.

To investigate the role of cardiomyocyte-expressed LRP6 in cardiac dysfunction, we generated tamoxifen-inducible, cardiac-specific LRP6-overexpressing mice. LRP6^CAG^ mice were bred with α-myosin heavy chain (α-MHC) Mer-Cre-Mer Tg mice (α-MHC-MCM, termed MCM) to generate LRP6^CAG^/MCM mice, which were injected with tamoxifen to induce cardiomyocyte-specific LRP6 overexpression (Figure 1C). After tamoxifen injection, cardiac LRP6 was overexpressed; however, in kidney tissues, LRP6 expression was not altered in LRP6^CAG^/MCM mice compared with DMSO injected controls (Figure 1D). LRP5 level was not affected by LRP6 overexpression (Figure S2). LRP6 was sharply increased in cardiomyocytes (CMs) but not in non-CMs from tamoxifen-injected-LRP6^CAG^/MCM compared to tamoxifen-injected-MCM hearts (Figure 1E).

We observed baseline cardiac geometry and function in tamoxifen-injected LRP6^CAG^ and MCM mice. About 8 weeks old male LRP6^CAG^ or MCM mice injected with tamoxifen had comparable left ventricular wall thickness, left ventricular inner dimension, ejection fraction (EF), fraction shortening (FS), heart rate (HR), and the ratio of heart weight/body weight (HW/BW) (Table S3-4). In this study, tamoxifen-injected MCM (termed MCM) mice were as control.

Two weeks after tamoxifen injection, LRP6^CAG^/MCM (marked LRP6 Over) or MCM male mice were subjected to TAC or sham operation (Figure 1F). LRP6 Over or MCM mice developed similar increases in left ventricular systolic pressure (LVSP) at 4 weeks post-TAC. The echocardiographic and hemodynamic assessment showed deterioration of myocardial contractile function (EF, FS, and dp/dt) and diastolic function (-dp/dt, LVEDP) accompanied by an increased left ventricular diastolic inner dimension (LVID; d) in MCM mice at 4 weeks after TAC compared with sham-operated MCM mice (Figure 1G, S3). These functional alterations in MCM mice were greatly attenuated in LRP6 Over mice post-TAC. Moreover, there were no differences in these parameters between MCM and LRP6 Over sham-operated mice (Figure 1G, S3).

These results suggested that cardiac overexpression of LRP6 protects the heart against pressure overload.

### Cardiomyocyte-expressed LRP6 inhibits cardiac hypertrophy and fibrosis induced by pressure overload

**Figure 2:**
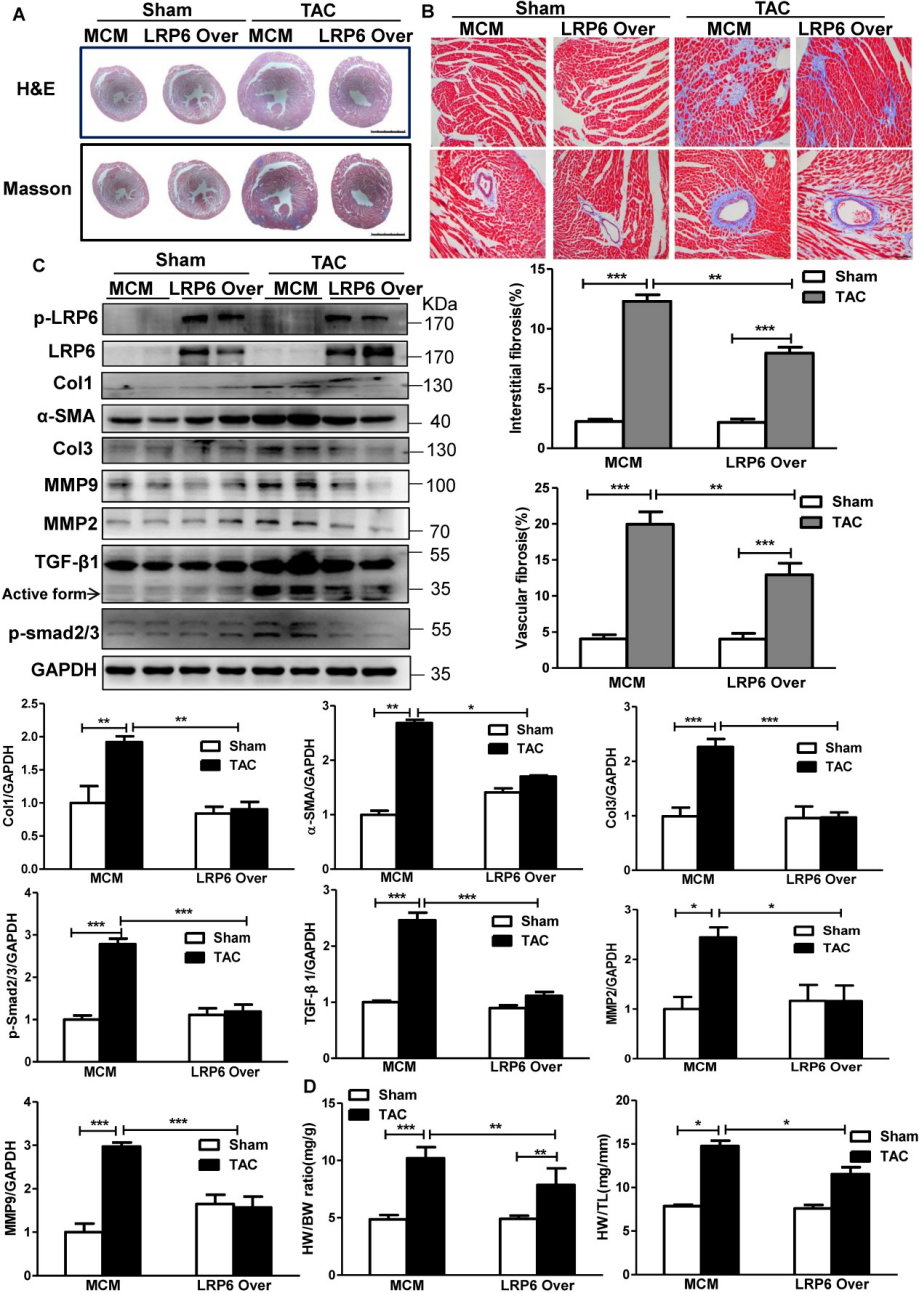
** Cardiomyocyte-specific overexpression of LRP6 attenuates cardiac hypertrophy and fibrosis induced by pressure overload.** (**A**), HE and Masson staining of cross heart sections. Scale bars, 2 mm. (**B**), Representative images of Masson staining heart tissue were showed and the cardiac interstitial and vascular fibrosis were quantitatively analyzed. Scale bars, 100 um. (**C**), Western blot analysis of p-LRP6, LRP6, Col1, Col3, α-SMA, MMP2, MMP9, TGF-β1, p-smad2/3 in heart tissues. *p < 0.05; **p < 0.01; ***p<0.001; n = 3-5/each group. (**D**), Quantitative analysis of the ratio of heart weight/body weight (HW/BW) and heart/tibia (HW/TL). *p < 0.05; **p < 0.01; ***p < 0.001; n = 4-7/each group. Tamoxifen-injected-LRP6^CAG^/MCM (LRP6 Over) or -MCM (MCM) mice were analyzed at 4 weeks after TAC or sham operation.

The pressure overload induced robust cardiac hypertrophy and fibrosis at 4 weeks after TAC in MCM mice, compared with the sham-operated MCM group (Figure 2A). Cardiomyocyte-specific LRP6 overexpression led to decreased interstitial and vascular fibrosis relative to MCM control mice at 4 weeks post-TAC (Figure 2A-B). Further analysis revealed that cardiac LRP6 overexpression induced increased p-LRP6 level in sham or TAC mice and inhibited the increased Col1 and Col3 protein levels induced by TAC (Figure 2C). TGF-β1, a critical pro-fibrotic factor, and the target pathway, p-Smad2/3, showed similar results (Figure 2C). The activation of MMP9 participates in TGF-β-induced tissue remodeling and fibrosis 18. The data showed that the MMP9 and *alpha*-smooth muscle actin (α-SMA, one of fibroblast activation markers) expression were greatly upregulated in MCM mice post-TAC, and the effects were attenuated in cardiac LPR6 overexpressing mice (Figure 2C). Immunofluorescence analysis revealed that TAC induced an increase in individual α-SMA+ cells in left ventricular tissue exclusive of vessel area, and cardiac LRP6 overexpression attenuated the effect (Figure S4). Also, cardiomyocyte-specific LRP6-overexpressing mice inhibited hypertrophic response compared to MCM control mice with lower HW/BW and HW/TL ratio, and smaller cardiomyocyte cross-area (CSA) (Figure 2D, S5).

These data indicated cardiomyocyte-expressed LRP6 inhibited cardiac hypertrophy and fibrosis induced by pressure overload.

### LRP6 regulates cardiomyocyte-derived-Wnt5a/11 under pressure overload

**Figure 3:**
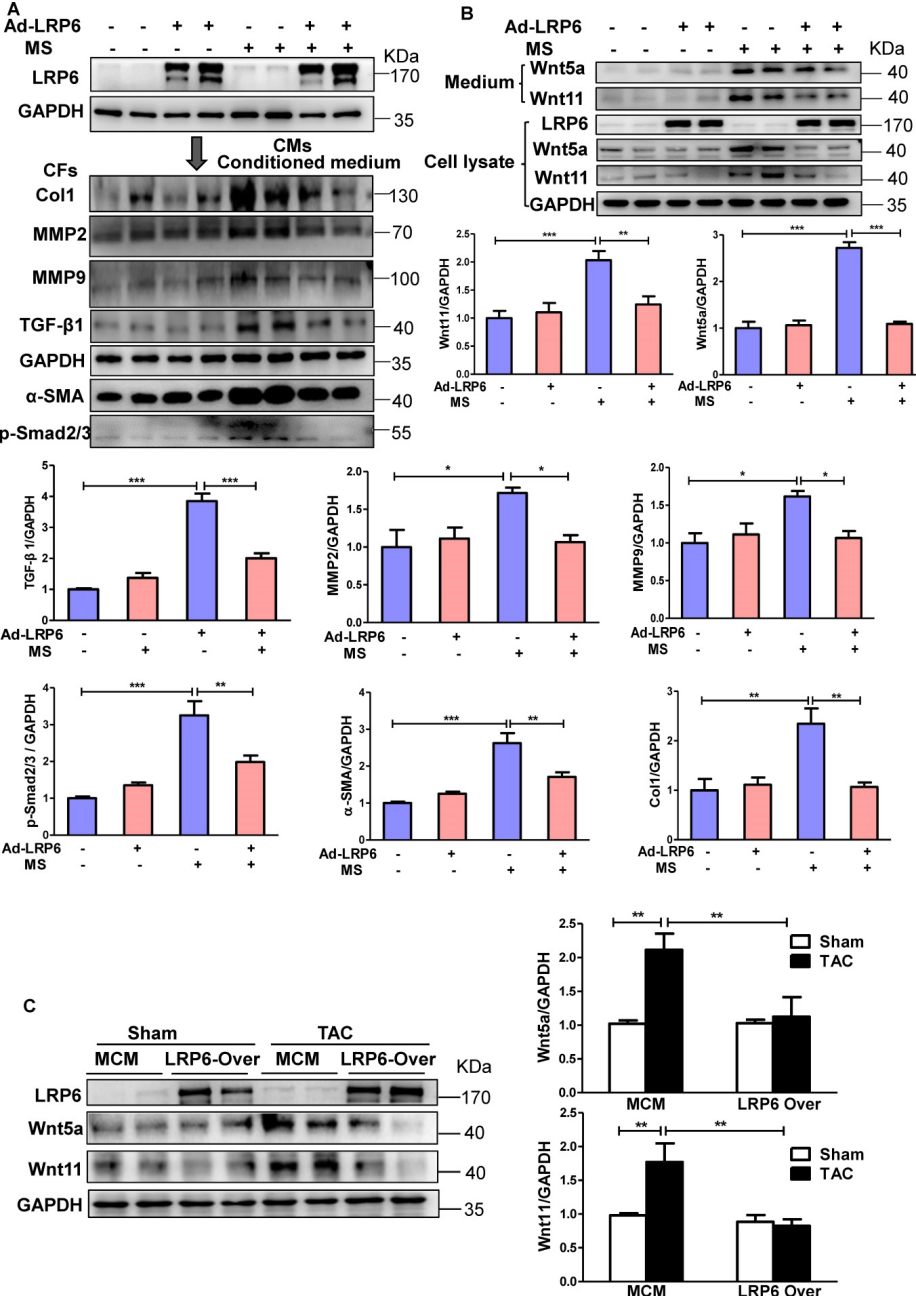
** Cardiomyocyte-specific overexpression of LRP6 inhibits the expression of cardiomyocyte-derived-Wnt5a/11 under pressure overload.** (**A**), Western blot analysis of LRP6 expression in CMs, and Col1, MMP2, MMP9, TGF-β1, α-SMA, and p-smad2/3 expression in CFs. Stretched (MS) or control CMs were pre-transfected with LRP6 adenovirus (Ad-LRP6) or control adenovirus (Ad-CON), and the culture medium from these CMs was used to stimulate CFs. *p < 0.05; **p < 0.01; ***p < 0.001; n = 4/each group. (**B**), Western blot analysis of Wnt5a and Wnt11 expression in CMs and the culture medium. Stretched (MS) or control CMs were pre-transfected with LRP6 adenovirus (Ad-LRP6) or control adenovirus (Ad-CON). **p < 0.01; ***p < 0.001; n = 5-6/each group. (**C**), Western blot analysis of Wnt5a and Wnt11 expression in tamoxifen-injected-LRP6^CAG^/MCM (LRP6 Over) or -MCM (MCM) mice after TAC or sham operation. **p < 0.01; n = 5/each group.

We subjected cultured CMs to mechanical stretch (MS) *in vitro* to mimic the *in vivo* pressure overload (Figure S6A). LRP6 adenovirus was transfected into CMs to induce LRP6 overexpression. When the cardiac fibroblasts (CFs) were stimulated with the conditioned medium from stretched CMs, Col 1, MMP2, MMP9, and TGF-β1 expression were increased, but this effects were not observed with the conditioned medium from LRP6-overexpressing CMs under mechanical stress (Figure 3A). The data suggested that LRP6 regulated cardiomyocyte-derived paracrine signaling to inhibit pressure overload-induced cardiac fibrosis.

The activation of β-catenin was reported to be involved in pressure overload-mediated cardiac hypertrophy 19 and fibrosis 20. LRP6, as a Wnt co-receptor, is involved in β-catenin regulation 2, and its cardiac-specific overexpression *in vivo* attenuated TAC-induced β-catenin activation (Figure S6B). *In vitro*, the mechanical stress-activated β-catenin, which was significantly inhibited by LRP6 overexpression in CMs (Figure S6C), could be related to the inhibitory effect of hypertrophy by LRP6 overexpression under pressure overload. However, the active β-catenin expression did not show any difference in cultured CFs treated with the conditioned medium from controls or LRP6-overexpressing CMs with or without mechanical stress (Figure S6C).

It is plausible that cardiomyocyte-specific LRP6 overexpression regulates the non-canonical Wnt secretion from CMs to inhibit pressure overload-mediated fibrosis. Wnt5a and Wnt11 usually act through the non-canonical pathway 21 and are involved in fibrosis development 22-24. *In vitro*, mechanical stress promoted the expression and secretion of Wnt5a and Wnt11 in cultured CMs, and this effect was attenuated by LRP6 overexpression (Figure 3B). We also examined the expression of Wnt5a and Wnt11 in cultured CFs stimulated with the conditioned medium from LRP6-overexpressing or control CMs with or without mechanical stress. Wnt5a and Wnt11 expression showed no difference between these groups (Figure S7A). *In vivo,* at 4 weeks after TAC, cardiac LRP6 overexpression significantly inhibited increased Wnt5a and Wnt11 protein levels without altering the mRNA levels (Figure 3C, S7B).

These data indicated that cardiomyocyte-specific LRP6 overexpression greatly suppressed the expression and secretion of Wnt5a and Wnt11 from stretched CMs, likely contributing to the inhibition of cardiac fibrosis under pressure overload.

### Wnt5a and Wnt11 are involved in cardiac fibrosis during pressure overload

**Figure 4:**
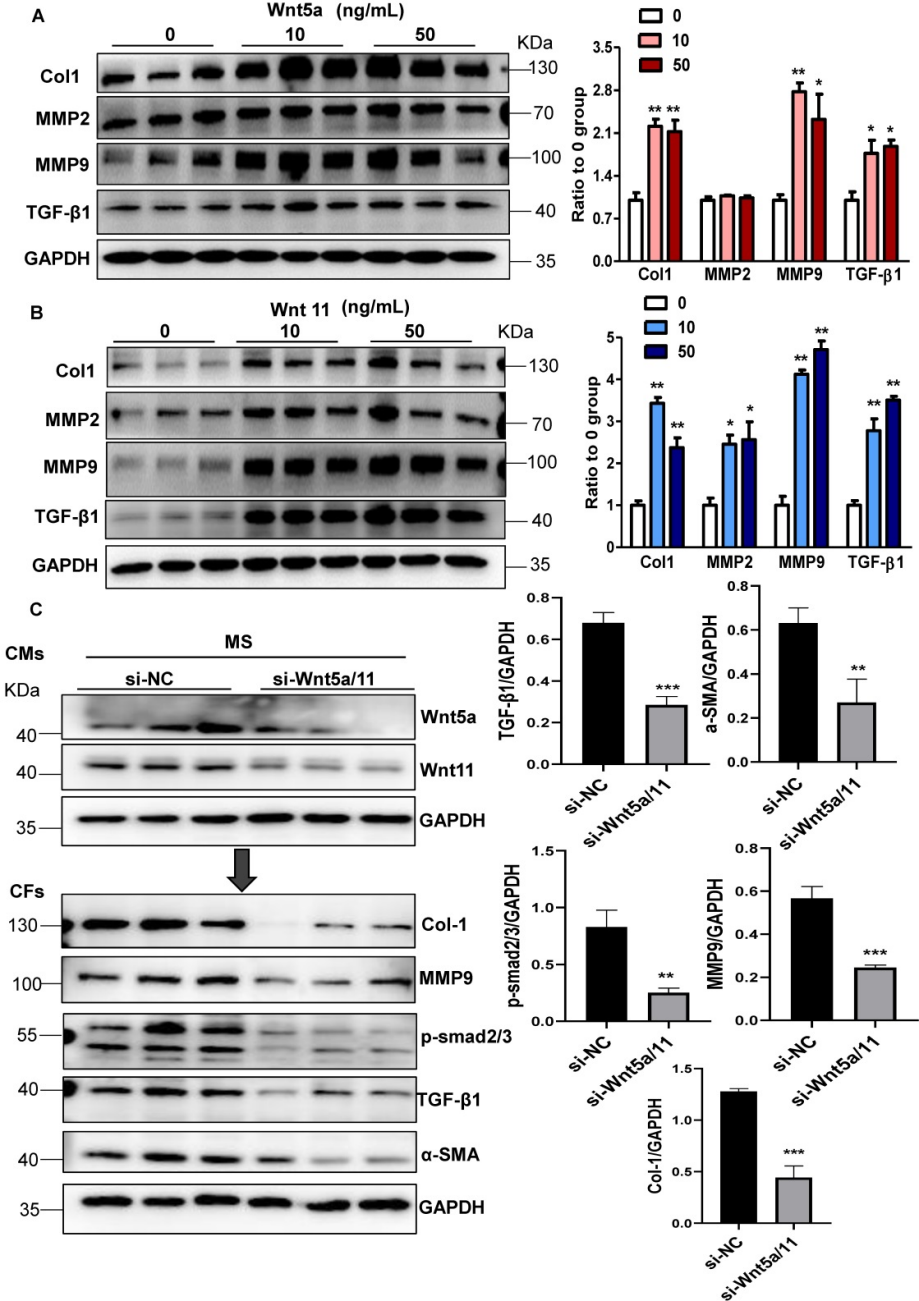
** Wnt5a and Wnt11 are involved in cardiac fibrosis during pressure overload.** (**A-B**), Western blot analysis of Col1, MMP2, MMP9, and TGF-β1 expression in CFs treated with Wnt5a or Wnt11 at 0, 10, or 50 ng/mL. *p < 0.05; **p < 0.01; n = 3/each group. (**C**), Western blot analysis of Wnt5a and Wnt11 in stretched CMs pre-transfected with si-Scramble (sh-NC) or si-Wnt5a plus si-Wnt11 (si-Wnt5a/11). The conditioned medium from the two groups of CMs was used to treat CFs for 24 h. Col1, MMP9, TGF-β1, α-SMA, and p-smad2/3 were analyzed by Western blotting. **p < 0.01; ***p < 0.001. n = 3/each group.

Next, we explored whether Wnt5a and Wnt11 were involved in cardiac fibrosis during pressure overload. In cultured CFs, Wnt5a or Wnt11 induced an increase in the expression of Col1, MMP9, and TGF-β1 at 10 ng/mL and 50 ng/mL (Figure 4A-B), but Wnt5a and/or Wnt11 did not activate β-catenin at 10ng/mL (Figure S8) indicating that Wnt5a or Wnt11 activated CFs by non-canonical Wnt signaling. We investigated whether cardiomyocyte-derived Wnt5a and Wnt11 activated CFs. siRNA-Wnt5a and siRNA-Wnt11 (si-Wnt5a/11) were transfected into CMs before mechanical stretch. The results revealed that si-Wnt5a/11 effectively knocked down the expression of Wnt5a and Wnt11 in stretched CMs. The Col1, MMP9, MMP2, and TGF-β1 levels were lower in CFs treated with the conditioned medium from stretched CMs transfected with siRNA-Wnt5a/11 than in control CFs treated with conditioned medium from stretched CMs (Figure 4C).

**Figure 5:**
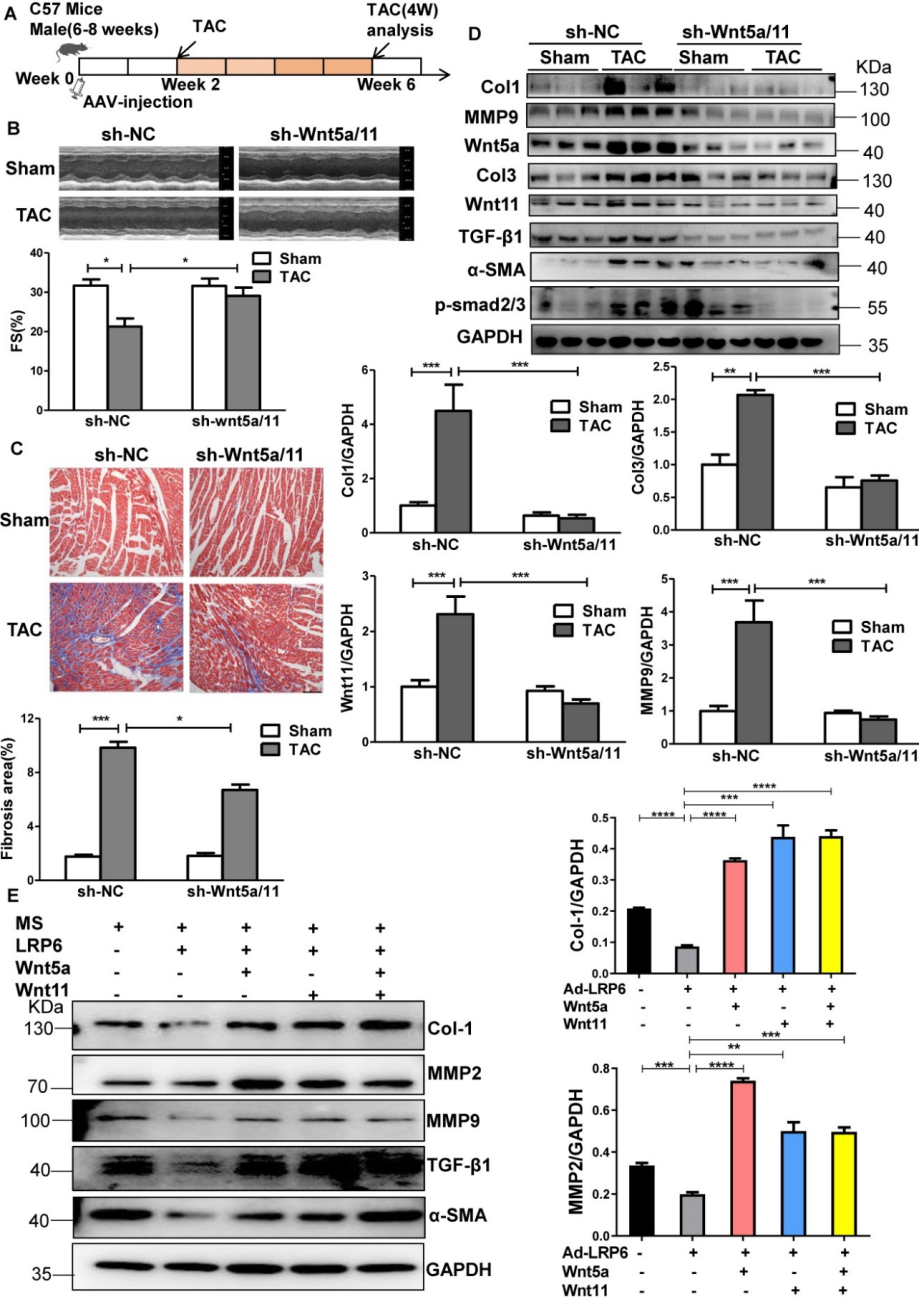
** Knockdown of Wnt5a and Wnt11 inhibits cardiac fibrosis in mice under pressure overload.** (**A**), Overall strategy to explore cardiac remodeling after knockdown of Wnt5a and Wnt11 in mice under pressure overload. (**B**), Fraction shortening (FS) in mice at 4 weeks after TAC. Mice were injected with sh-Wnt5a/Wnt11-AAV9 (sh-Wnt5a/Wnt11) or sh-scramble-AAV9 (shNC) by tail vein two weeks before TAC or sham operation. *p < 0.05; n = 6-10/each group. (**C**), Masson staining of heart tissue from mice in (B) and fibrosis area was quantitatively analyzed. Bar = 100 µm. *p < 0.05; ***p < 0.001. n = 4-9/each group. (**D**), Western blot analysis of Wnt5a, Wnt11, Col1, Col3, MMP9, TGF-β1, α-SMA, and p-smad2/3 expression in heart tissue from TAC or sham mice pre-injected with sh-Wnt5a/Wnt11-AAV9 (sh-Wnt5a/Wnt11) or sh-scramble-AAV9 (sh-NC) by tail vein. **p < 0.01; ***p < 0.001. n = 4-6 mice/each group. (E), Western blot analysis of Col1, MMP2, MMP9, TGF-β1 and α-SMA expression in CFs stimulated with the culture medium from control (Ad-CON) or LRP6 overexpressing CMs (Ad-LRP6), Wnt5a, Wnt11, Wnt5a+Wnt11 at 10 ng/mL, or PBS were supplemented with the culture medium from Ad-LRP6-CMs under MS and treated with CFs. *p < 0.05; **p < 0.01; ***p < 0.001. n = 3/each group.

The Wnt5a/11-AAV9 shRNAs were injected into C57BL/6 mice two weeks before sham-operation or TAC for the *in vivo* knockdown of the cardiac Wnt5a and Wnt11 expression; sh-scramble-AAV9 was injected as a control (Figure 5A). At 4 weeks post TAC, the shRNA-Wnt5a/11-AAV9 injection greatly improved cardiac function with higher FS than shRNA-scramble injection (Figure 5B), and suppressed cardiac fibrosis evidenced by the lower fibrosis area, and decreased Col1, Col3, MMP9 and TGF-β1 expression (Figure 5C-D, S9A).

*In vitro,* the conditioned medium from LRP6-overexpressing CMs under stretch induced decreased expressions of Col1, MMP2, MMP9, TGF-β1, and α-SMA compared with the conditioned medium from control CMs under stretch. The effects were reversed by supplementing Wnt5a, Wnt11, or Wnt5a+Wnt11 in CFs (Figure 5E, S9B). However, Wnt5a plus Wnt11 did not promote the rescue effects compared with either Wnt5a or Wnt11 groups.

These data suggested that cardiomyocyte-specific LRP6 overexpression inhibited cardiac fibrosis due to the decreased expression and secretion of Wnt5a or Wnt11 from CMs under pressure overload.

### LRP6 regulates Wnt5a/11 by Cythepsin D in cardiomyocytes under pressure overload

**Figure 6:**
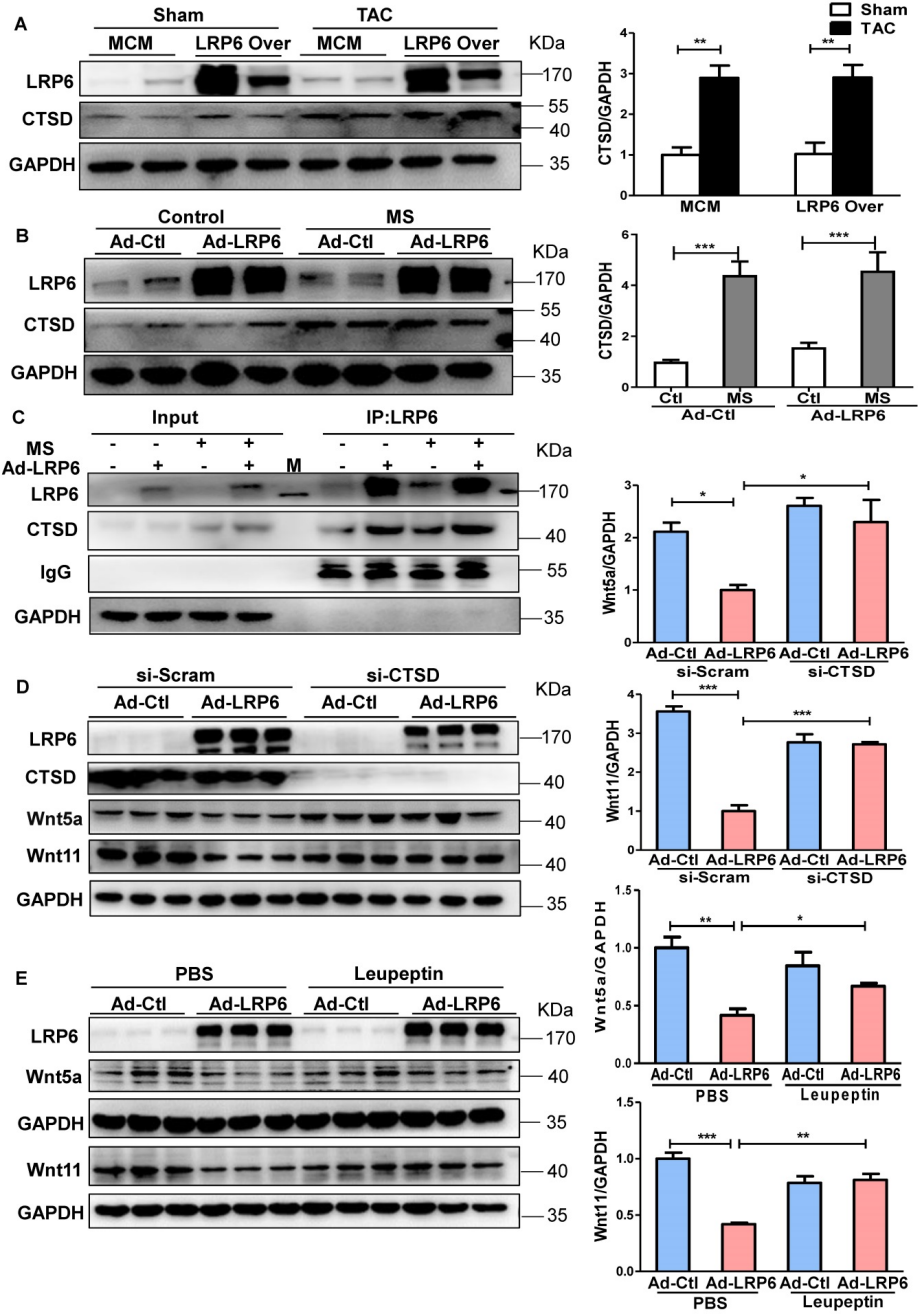
** Cardiomyocyte-specific overexpression of LRP6 promotes the degradation of Wnt5a and Wnt11 by interaction with LRP6 and CTSD during pressure overload.** (**A**), Western blot analysis of LRP6 and CTSD expression in tamoxifen-injected LRP6^CAG^/MCM (LRP6 Over) or -MCM (MCM) mice at 4 weeks after TAC or sham operation. *p < 0.05; **p < 0.01; n = 4 mice/each group. (**B**), Western blot analysis of LRP6 and CTSD expression in control (Ad-CON) or LRP6 overexpressing CMs (Ad-LRP6) with or without MS. ***p < 0.001; n = 4-6/each group. (**C**), Interaction of LRP6 and CTSD was analyzed by IP analysis in control (Ad-CON) or LRP6-overexpressing CMs (Ad-LRP6) with or without MS. M: marker. (**D**), Western blot analysis of Wnt5a, Wnt11, and CTSD expression in stretched control (Ad-CON) or LRP6 overexpressing CMs (Ad-LRP6) after treatment with si-CTSD or si-scramble. *p < 0.05; ***p < 0.001; n = 3/each group. (**E**), Western blot analysis of Wnt5a and Wnt11 expression in stretched control (Ad-CON) or LRP6-overexpressing CMs (Ad-LRP6) after treatment with PBS or leupeptin (50 µg/ml). *p < 0.05; **p < 0.01; ***p < 0.001; n = 3 /each group.

To explore the potential mechanisms, we analyzed the proteins associated with LRP6 by Nano-HPLC-MS/MS analysis in LRP6-overexpressing CMs under stretch (Figure S10A). The proteins were separated, the gels were silver-stained, and the darkest bands of about 45 kDa in stretched LRP6-overexpression group compared with the control group were chosen for further analysis. The data showed 22 proteins associated with LRP6-overexpressing CMs under mechanical stress (Table S5). The heat shock cognate 71 kDa protein (HSP7C) was detected in the 45 kDa region, probably as the degradation or cleaved form. Among these proteins, cathepsin D (CTSD) (Figure S10B, Table S6), an aspartyl protease, was of interest as it is expressed in the lysosome, endosome, extracellular space, and membrane raft 25-27. The 45 kDa pro-CTSD is an intermediate form of CTSD that yields the CTSD mature form. TAC enhanced the expression and activity of CTSD, but LRP6 overexpression did not alter the CTSD effects in TAC mice (Figure 6A, S10C). The results in stretched CMs were consistent with the *in vivo* observations (Figure 6B, S10C). IP analysis revealed that LRP6 overexpression increased the association between LRP6 and CTSD in stretched CMs compared with the controls (Figure 6C). To explore whether CTSD is required for the degradation of Wnt5a or Wnt11, we knocked down CTSD expression by siRNA transfection in CMs (Figure 6D, S11) and found that si-CTSD attenuated the inhibition of Wnt5a or Wnt11 expression induced by LRP6 overexpression in stretched-CMs (Figure 6D). We then treated CMs with leupeptin, a protease inhibitor, which also inhibits CTSD maturation and observed the similar results of Wnt5a and Wnt11 expression in stretched CMs (Figure 6E).

From these data, we speculated LRP6 overexpression promoted the complex formation of LRP6 and CTSD, promoting the degradation of Wnt5a and Wnt11 by CTSD during pressure overload.

### Protease inhibitor attenuates LRP6/CTSD interaction and enhances cardiac fibrosis in cardiomyocyte-specific LRP6-overexpressing mice under pressure overload

**Figure 7:**
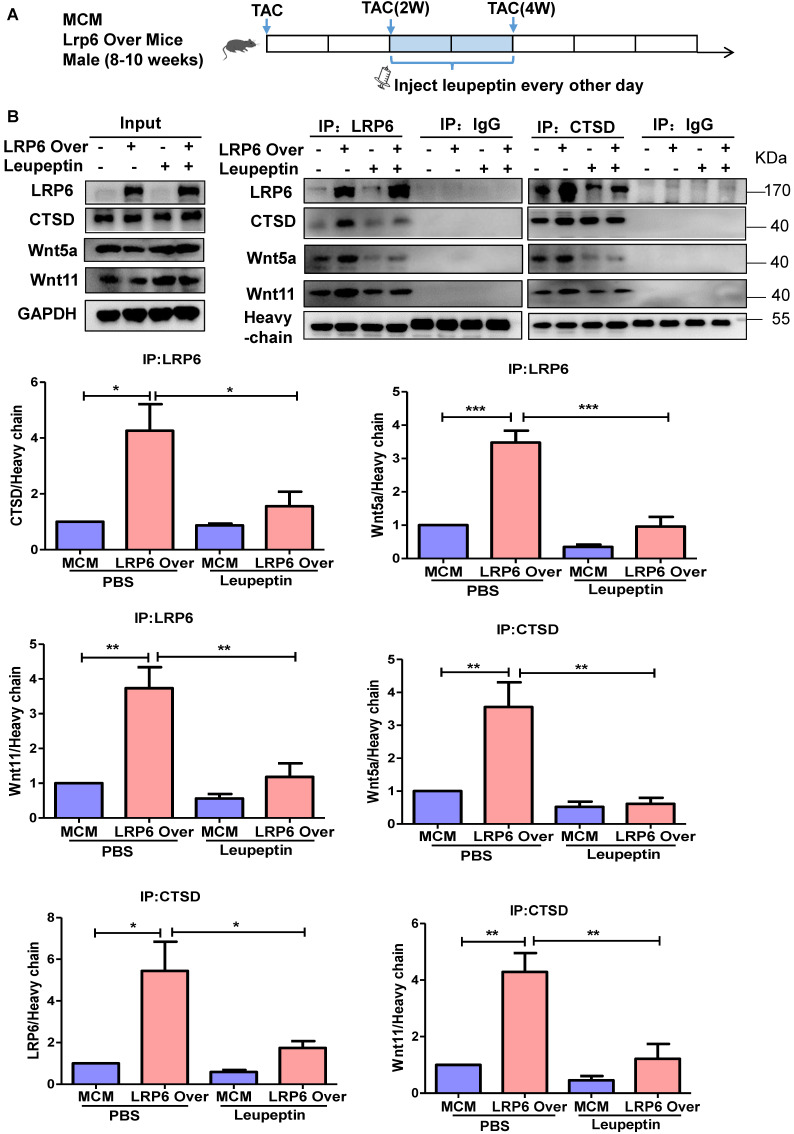
** Protease inhibitor attenuates the interaction of LRP6 and CTSD in cardiomyocyte-specific LRP6-overexpressing mice under pressure overload.** (**A**), Schematic of the experimental procedure in (B). A protease inhibitor, leupeptin (40 mg/kg), was intraperitoneally injected into LRP6 Over or -MCM mice from 2 to 4 weeks (every other day) after TAC; PBS treatment was used as a control. (**B**), IP analysis of the interaction among LRP6, CTSD, Wnt5a, and Wnt11. The experiment was repeated for at least 3 times.

**Figure 8:**
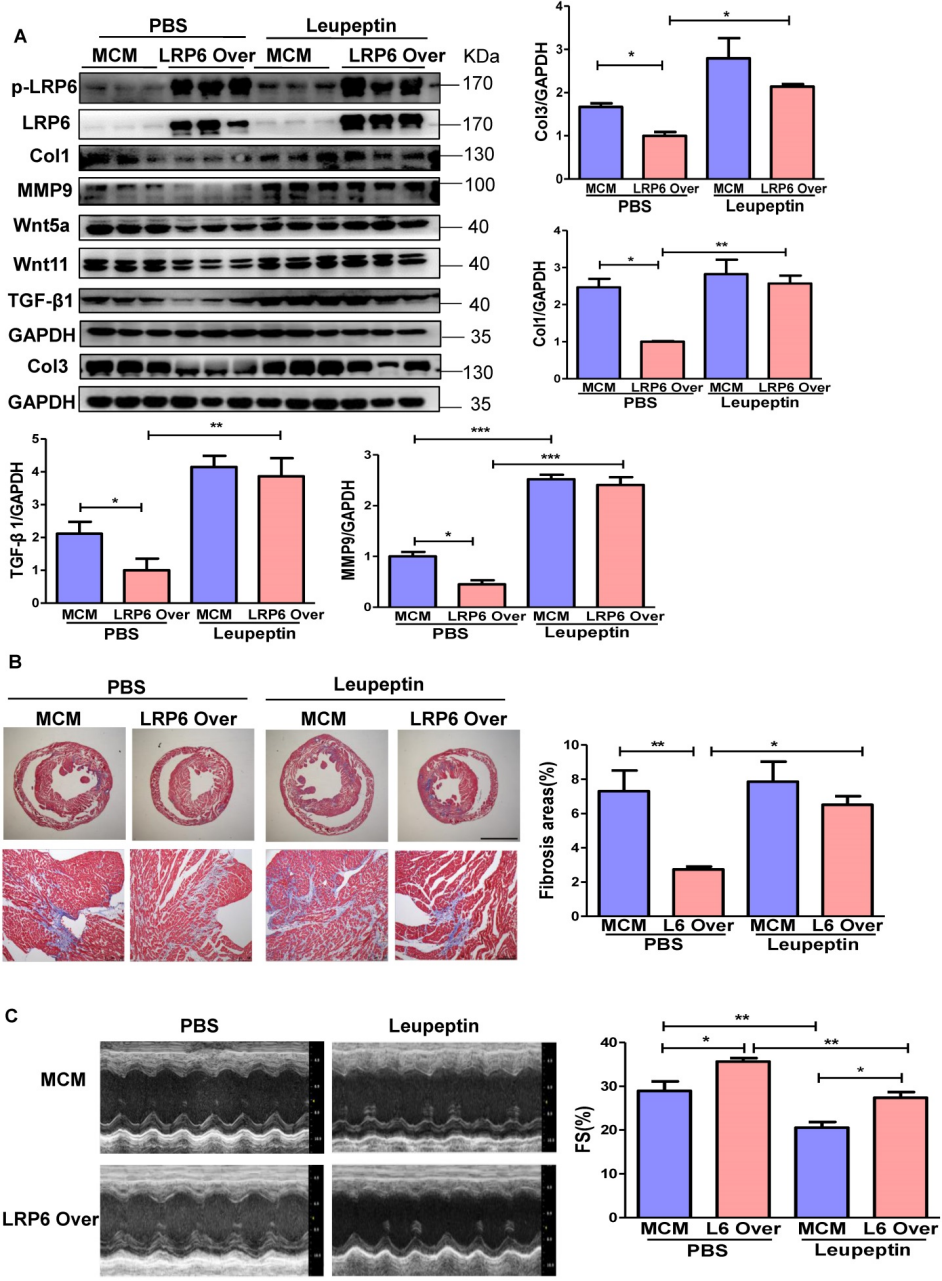
** Protease inhibitor attenuates cardiac protection in cardiomyocyte-specific LRP6-overexpressing mice under pressure overload.** (**A**), Western blot analysis of p-LRP6, LRP6, Wnt5a, Wnt11, Col1, Col3, MMP9 and TGF-β1 in the heart tissue. *p < 0.05; **p < 0.01; ***p < 0.001; n = 3/each group. (**B**), Masson staining of heart tissue and quantitative analysis of fibrosis area. The upper lane: bar = 2 mm; The lower lane: bar = 100 µm, *p < 0.05; **p < 0.01; n = 7-10 /each group. (**C**), Fraction shortening (FS) in mice. *p < 0.05; **p < 0.01; n = 8/ each group.

LRP6 overexpression promoted the interaction between LRP6 and CTSD and decreased Wnt5a or Wnt11 expression in left ventricular tissues at 4 weeks post-TAC. We treated mice with leupeptin 2 weeks post-TAC (Figure 7A) and observed that leupeptin greatly decreased the interaction between CTSD (intermediate form) and LRP6, and attenuated the decreased expression of Wnt5a and Wnt11 in LRP6-overexpressing hearts at 4 weeks after TAC (Figure 7B, Figure 8A, S12A). Leupeptin treatment reversed cardiac fibrosis inhibition as evidenced by lager fibrosis area and higher expression of Col1, Col3, MMP9, MMP2, and TGF-β1 than the control PBS treatment in LRP6 overexpressing hearts after TAC (Figure 8A, 8B). The echocardiographic analysis showed that leupeptin treatment greatly suppressed FS increase induced by cardiac overexpression of LRP6 in mice at 4 weeks after TAC (Figure 8C). However, leupeptin did not alter the fibrosis but deteriorated cardiac function in MCM mice post-TAC (Figure 8A-C). It also did not affect HW/BW and HW/TL in LRP6-overexpressing or control mice post-TAC (Figure S12B-C). The results indicated that leupeptin showed few cardiac hypertrophy effects in LRP6-overexpressing hearts or the MCM group after TAC.

These data suggested that the protease inhibitor attenuated cardiac fibrosis inhibition induced by LRP6 overexpression due to the decreased degradation of Wnt5a and Wnt11 in CMs of LRP6-overexpressing mice after TAC.

### Mutations in LRP6 or CTSD inhibit their interaction

**Figure 9:**
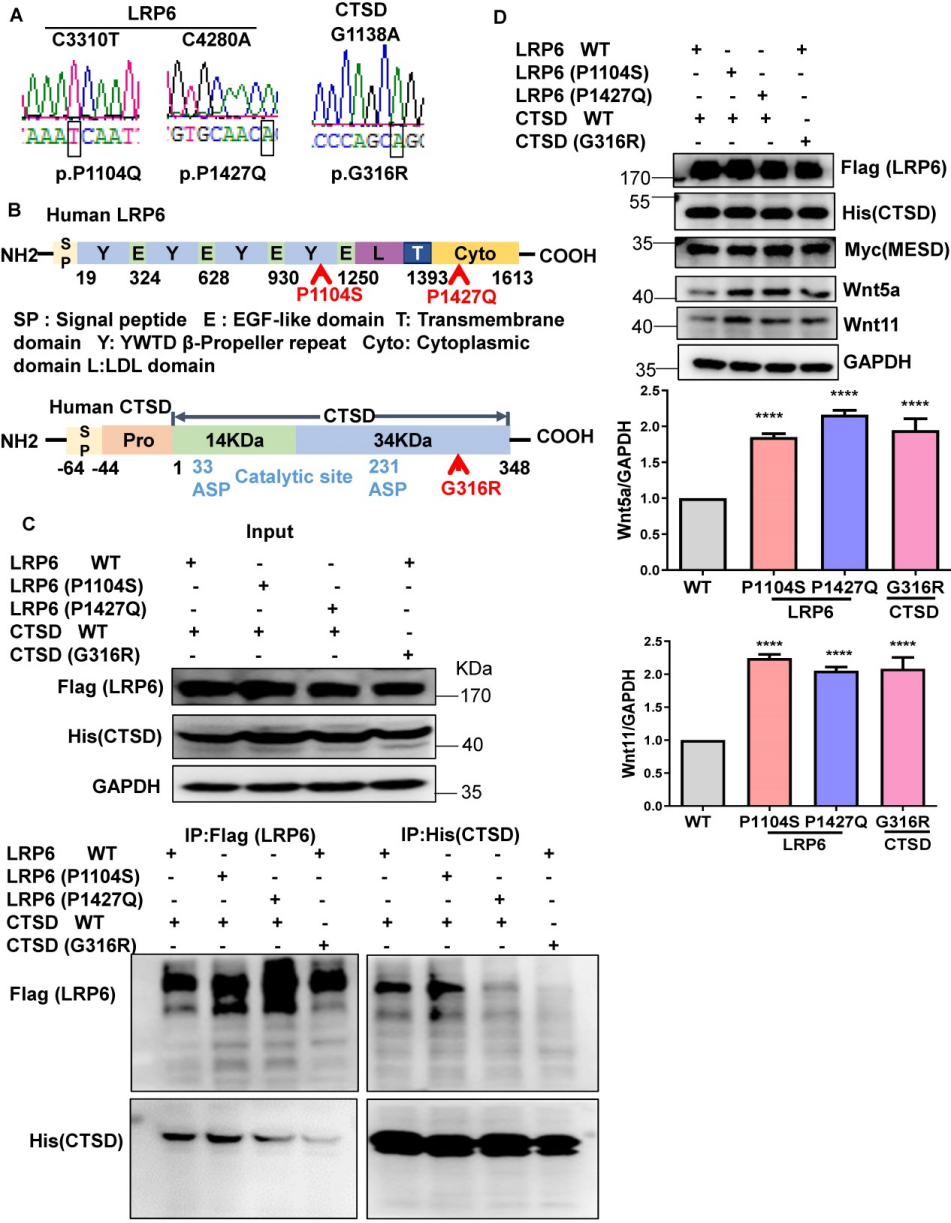
** Mutants of LRP6 or CTSD inhibit the interaction of LRP6 and CTSD.** (**A**), Sequencing results of LRP6 and CTSD mutations. (**B**), Schematic diagram of LRP6 and CTSD showing approximate locations of the mutants. (**C**), IP analysis of the interaction among LRP6 WT, LRP6 (P1104S), or LRP6 (P1427Q) and CTSD WT or CTSD (G136R). LRP6 WT and CTSD WT or their mutant plasmids were transfected into 293T cells for 48 h. The experiment was repeated 4 times. (**D**), Western blot analysis of LRP6, CTSD, Wnt5a, Wnt11, and MESD in stretched COS7 cells transfected with LRP6 WT and CTSD WT or their mutant plasmids as in (C). ^****^P < 0.0001, n = 3/each group.

To explore the clinical significance of the interaction between LRP6 and CTSD in cardiac remodeling, we constructed the mutants of human LRP6 and CTSD (LRP6^C3301T^ (p.P1104S), LRP6^C4280A^ (p.P1427Q), and CTSD^G1138A^ (p.G316R). LRP6^C3301T^ and CTSD^G1138A^ were found in dilated cardiomyopathy (DCM) patients with hypertension but not in control patients. The basic characteristics of these patients are listed in Table S7. LRP6^C4280A^ has been reported in a previous study 28. We constructed these mutants and confirmed the mutations by sequence analysis (Figure 9A). The corresponding sites of the mutants are marked in the amino sequence from LRP6 and CTSD (Figure 9B). The MESD plasmid was transfected into 293 cells with the LRP6 plasmid to facilitate membrane localization of LRP6. The WT and mutants of LRP6 or CTSD were effectively expressed in 293T cells with no obvious difference between WT and any of the mutants (Figure 9C). IP analysis revealed that LRP6 strongly interacted with CTSD, and the interaction was dramatically attenuated by LRP6 (P1427Q) or CTSD (G316R) but was not affected by LRP6 (P1104S) (Figure 9C). The CTSD band at about 48 kDa in Figure 9C likely represents the CTSD intermediate form.

We also examined Wnt5a and Wnt11 expression in stretched COS7 cells transfected with LRP6 or CTSD WT plasmids and their mutants. The WT and mutants of LRP6 or CTSD and MESD were effectively expressed in COS7 cells under mechanical stretch. Also, Wnt5a and Wnt11 expression increased in LRP6 or CTSD mutants compared with LRP6 and CTSD WT groups (Figure 9D).

These data suggested that the increased expression of Wnt5a and Wnt11 might partly be owing to the decreased interaction between LRP6 and CTSD, involved in pathological cardiac conditions under pressure overload.

## Discussion

**Figure 10:**
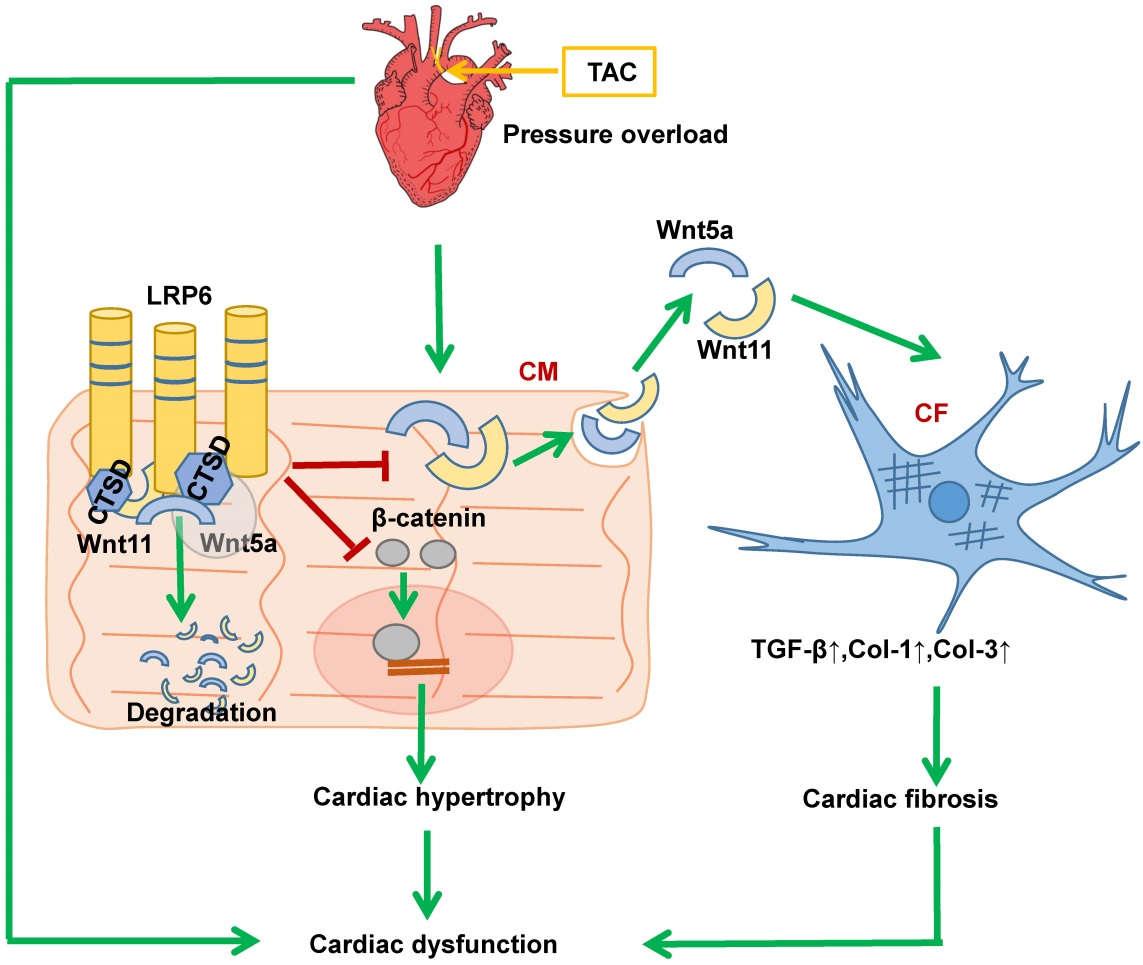
** Proposed model of cardiac protection mediated by cardiac-specific LRP6 overexpression in response to pressure overload.** LRP6 overexpression suppresses β-catenin activation, which may contribute to the inhibition of cardiac hypertrophy. LRP6 overexpression inhibits cardiomyocyte-derived Wnt5a/Wnt11 to inhibit cardiac fibrosis. Cardiomyocyte-expressed LRP6 interacts with CTSD, which may promote the degradation of Wnt5a and Wnt11.

Our findings demonstrate the ability of cardiomyocyte-expressed LRP6 to mediate cardiac protection in response to pressure overload. Significantly, we observed that cardiac-specific LRP6 overexpression inhibited cardiac fibrosis by decreased expression and secretion of Wnt5a and Wnt11 by regulating CTSD in CMs under pressure overload. The association of LRP6 with CTSD may be critical for the degradation of Wnt5a and Wnt11, as knockdown of CTSD or treatment with protease inhibitor reduced Wnt5a and Wnt11 degradation in LRP6-overexpressing CMs under pressure overload. We provided evidence that LRP6 functioned as a regulator of CTSD, Wnt5a and Wnt11 in the heart under pressure overload (Figure 10). Furthermore, our study revealed that LRP6 inhibited β-catenin activation, which may be related to the reduced cardiac hypertrophy in LRP6-overexpressing mice under pressure overload (Figure 10). Our findings, however, do not exclude the possibility that other LRP family members may be involved in signaling pathways in the cardiac pathophysiological response.

Our recent study indicated that LRP6 knockout inhibited autophagic degradation 9. Cardiac LRP6 overexpression inhibited cardiac hypertrophy and fibrosis but did not promote autophagy after pressure overload (data not shown). The activation of β-catenin is involved in cardiac hypertrophy and fibrosis mediated by pressure overload 19, 20. LRP6 overexpression inhibited β-catenin activation in CMs, likely resulting in cardiac hypertrophy inhibition under pressure overload. Conditioned medium from LRP6-overexpressing CMs under mechanical stretch inhibited the activation of CFs but did not affect β-catenin expression. Therefore, we investigated the specificity of cardiomyocyte secretory proteins for LRP6 signaling in the heart. LRP6 is one of Wnt co-receptors and mediates canonical Wnt/β-catenin signaling, but whether it regulates the expression or secretion of Wnts, including non-canonical Wnts, is largely unknown. LRP6 overexpression inhibited the expression and secretion of Wnt5a and Wnt11 in CMs under mechanical stretch. Wnt5a and Wnt11 usually act through the non-canonical pathway 21. Wnt5a is elevated in the myocardium of heart failure patients and is associated with cardiac dysfunction 24, 29. It promotes myocardial inflammation and fibrosis by inducing interleukin (IL)-6 and tissue inhibitor of metalloproteinase (TIMP)-1^24^. Wnt11 promotes renal fibrosis by cooperating with TGF-β signaling through non-canonical Wnt signaling 23. The knockdown of Wnt5a and Wnt11 significantly inhibited cardiac fibrosis under pressure overload. These data revealed that LRP6 suppressed cardiomyocyte-derived Wnt5a/11 to inhibit cardiac fibrosis under pressure overload.

Pressure overload induces the interaction of LRP6 and CTSD, and LRP6 overexpression enhanced this association in stretched CMs. Since LRP6 overexpression did not alter the mRNA expression of Wnt5a and Wnt11 in heart tissue after pressure overload, we explored the possibility that LRP6 overexpression promoted the degradation of Wnt5a and Wnt11 by regulating CTSD in CMs. CTSD is a ubiquitously and abundantly expressed cathepsin. The human CTSD gene contains 9 exons, and the protein is synthesized in the rough endoplasmic reticulum as pre-pro-CTSD, including a signal peptide and the 52 kDa pro-CTSD. After removing the 44 amino acid N-terminal, the pro-peptide produces a 48 kDa single-chain intermediate, an active enzyme located in the endosomes, lysosomes or extracellular space. Further proteolytic cleavage yields the CTSD mature form composed of a 34 kDa heavy chain and a 14 kDa light chain 30-32. In the present study, the rodent CSTD peptide associated with LRP6 was identified to be about 45 kDa, shorter than 48 kDa, which might be due to the species difference.

CTSD exerts a critical role in the lysosomal degradation of autophagosomes involved in immune response, apoptosis, cancer development, and myocardial infarction 33-36. CTSD deficiency impairs myocardial autophagosome removal and results in restrictive cardiomyopathy 36. In our study, pressure overload induced increased expression and activity of CTSD, and LRP6 overexpression increased its interaction with CTSD. CTSD was previously demonstrated to cleave α-synuclein and decrease its toxicity in the mouse brain *in vivo* 37, 38. In the cultured CMs, knockdown of CTSD attenuated the inhibitory effects induced by LRP6 overexpression on the upregulation of Wnt5a or Wnt11 in stretched-CMs. These data suggested that CTSD was required for the degradation of Wnt5a and Wnt11 or other selective proteins in LRP6-overexpressing CMs under pressure overload. CTSD and proCTSD interact with many important molecules, such as FGF, IL-1, and myosin, to induce their degradation or participate in multiple processes, including apoptosis or cancer development 39. In the present study, LRP6 overexpression had few effects on CTSD activity after TAC. Hence, we speculated the association of LRP6 and CTSD might be critical for the degradation of Wnt5a and Wnt11 involved in myocardial remodeling during pressure overload.

Cysteine cathepsins, especially CTSB and CTSL, are involved in CTSD processing 30, 32. Leupeptin, a cathepsin inhibitor, also inhibits CTSD maturation 30, 40. In the present study, leupeptin attenuated the interaction between LRP6 and CTSD and inhibited LRP6-induced Wnt5a and Wnt11 degradation in CMs under pressure overload. Leupeptin treatment enhanced cardiac fibrosis and attenuated cardiac protection induced by LRP6 overexpression against pressure overload. However, leupeptin did not affect cardiac hypertrophy in LRP6-overexpressing mice under pressure overload. Leupeptin reduces protein degradation by inhibiting the proteolytic activity of some proteases in the heart or other tissues 40, 41. We could not exclude the possibility that leupeptin inhibited protein degradation by other proteases in LRP6-overexpressing mice under pressure overload. Wnt5a promotes liver fibrosis by FZD2 and FZD8 42, and Wnt11 binds to FZD8 known to be involved in TGF-β signaling in prostate cancer 43. It is possible that leupeptin also decreases the degradation of FZDs, enhancing their binding with Wnt5a or Wnt11 and FZDs and contributing to cardiac fibrosis. This could explain the observation that leupeptin caused decreased interaction between Wnt5a or Wnt11 and LRP6.

We explored the clinical significance of the LRP6 and CTSD interaction in cardiac remodeling by analyzing human LRP6 mutants (P1104S and P1427Q) and CTSD (G316R). LRP6 (P1104S) and CTSD (G316R) were verified in DCM patients with hypertension from Tongji Hospital (Wuhan, China). LRP6 (P1427Q) mutation increases β-catenin signaling, reported in a previous study 28. The present data showed that LRP6 (P1427Q) and CTSD (G316R), but not LRP6 (P1104S), greatly suppressed the interaction of LRP6 and CTSD. Under mechanical stretch, Wnt5a or Wnt11 was higher in either LRP6 (P1104S), LRP6 (P1427Q) or CTSD (G316R) group than WT group. These data suggested that the increased Wnt5a and Wnt11 levels were partly from the decreased interaction of CTSD and LRP6. P1104 is located near the transmembrane region extracellular domain of LRP6. CTSD was also expressed in the extracellular matrix of cells, the interaction between LRP6 and CTSD was also likely in the extracellular space of CMs. The LRP6 (P1104S) mutation didn't affect the interaction with CTSD, but it could inhibit the interaction of Wnt5a or Wnt11 and LRP6, blocking the degradation of Wnt5a or Wnt11 by CTSD and resulting in pathological cardiac remodeling. Thus, it could explain the association of LRP6 (P1104S) mutation with DCM.

The P1427Q mutation is located in the intracellular domain near the phosphorylation motif of LRP6 and was identified in the Chinese Han Population with neural tube defects (NTD) and induced over-active Wnt/β-catenin signaling 28. However, it was not mentioned whether these patients carrying the mutation developed cardiac remodeling. Given that the other mutants of LRP6 (P1104S) and CTSD (G316R) in hypertension patients were associated with DCM, it is possible that the patients with LRP6 (P1427Q) develop cardiac remodeling or dysfunction in response to stress such as pressure overload or ischemia. Two key aspartic residues, Asp33 and Asp233 of CTSD, located on the 14kD and 34kD chains, respectively, were identified to be the catalytic sites of human CTSD 44. Although CTSD interacts with other proteins to induce protein degradation or affect other cellular processes, effective binding sites of CTSD have not been reported. In the present study, G316R CTSD mutant greatly inhibited the interaction between CTSD and LRP6. G316R is located near the carboxy-terminal domain of human CTSD and not in the catalytic site, which might be critical for CTSD binding to LRP6 or other proteins. In the future, the functional role of mutants needs to be explored in CMs.

In conclusion, cardiomyocyte-expressed-LRP6 regulated CTSD, promoted the degradation of Wnt5a and Wnt11, and inhibited their secretion from CMs, ameliorating cardiac fibrosis and preventing maladaptive cardiac remodeling under pressure overload. LRP6 may serve as a novel therapeutic target for abrogating maladaptive cardiac remodeling and heart failure.

## Supplementary Material

Supplementary figures and tables.

## Abbreviations

LRP6: Low-density lipoprotein receptor-related protein 6
TAC: transaortic constriction
CAT: chloramphenicol acetyltransferase
CTSD: cathepsin D
DCM: dilated cardiomyopathy
MCM: α-myosin heavy chain Mer-Cre-Mer
FS: fraction shortening
EF: ejection fraction
HR: heart rate
LV: left ventricle
RIPA: radioimmunoprecipitation assay
HE: hematoxylin-eosin
SD: Sprague-Dawley
AAV9: adeno-associated virus 9 vector
HW/BW: heart weight/body weight
HW/TL: heart/tibia length
LVSP: left ventricular systolic pressure
LVEDP: left ventricular end diastolic pressure
LVID: left ventricular diastolic inner dimension
TGF-β: transforming Growth Factor-beta
MMP: Matrix Metalloproteinase
α-SMA: alpha-smooth muscle cells
CSA: cross-section area
CMs: Cardiomyocytes
CFs: cardiac fibroblasts
MS: mechanical stretch
TIMP: tissue inhibitor of metalloproteinase
NTD: neural tube defects
