# Supplementary material for: Low-density lipoprotein receptor-related protein 6 regulates cardiomyocyte-derived paracrine signaling to ameliorate cardiac fibrosis
Source: Theranostics. 2021 Jan 1;11(3):1249–68. doi: 10.7150/thno.48787 (PMC7738902; doi:10.7150/thno.48787)
Supplement: Supplementary file 1 — Supplementary figures and tables. [file thnov11p1249s1.pdf]

Immunofluorescence staining

OCT-embedded heart tissue was cut into 5-µm sections for staining studies. Tissue sections were fixed in 4 % polyformaldehyde for 10-15 minutes at room temperature and washed three times with PBS for 5 min each. After blocking with 5% BSA for 1 hour at room temperature, washing with PBS was repeated three times for 5 minutes each time. The sections were incubated with the primary antibody at 4 ° C overnight. After the unbound primary antibody was washed with PBS, the sections were incubated in the secondary antibody (1: 1,000, Alexa Fluor 488/594, Thermo Fisher Scientific, Inc.) at 37 ° C for 1 hour and then were washed with PBS for three times. Nuclei were stained with 4',6-diamidino-2-phenylindole (DAPI, 1:5000 Sigma) at room temperature for 5 min. Finally tissue sections were mounted with coverslips. The confocal imaging systems (Zeiss, Germany) were used to capture images for analysis. Goat serum was used instead of primary antibody to stain section as control.

Table S1. Primers used in real-time PCR analysis.

**Gene name**	**Forward primer, 5'-3'**	**Reverse primer, 3'-5'**
Wnt5a (mouse)	CTGCGGAGACAACATCGACTA	CGTGGATTCGTTCCCTTTCTCTA
Wnt11 (mouse)	GCACTGAATCAGACGCAACAC	CGACAGGGCATACACGAAGG
GAPDH (mouse)	ACCACAGTCCATGCCATCAC	TCCACCACCCTGTTGCTGTA
MMP2 (mouse)	CAGGGAATGAGTACTGGGTCTATT	ACTCCAGTTAAAGGCAGCATCTAC
MMP9 (mouse)	AATCTCTTCTAGAGACTGGGAAGGAG	AGCTGATTGACTAAAGTAGCTGGA
Col1α1 (mouse)	CCTCAAGGGCTCCAACGAG	TCAATCACTGTCTTGCCCCA
Col3α1(mouse)	ACGTAGATGAATTGGGATGCAG	GGGTTGGGGCAGTCTAGTC
GAPDH (rat)	AACAAGCAACTGTCCCTGAGC	GTAGACAGAAGGTGGCACAGA
CTSD (rat)	CATCGCAGCCAAGTTTGATG	CCGGGAGCACATTGTTAACA

Table S2. All the primary antibodies used in the Western blot analysis.

Antibody	Company	Catalog#	Size(kDa)	2°
LRP6(C47E12)Rabbit mAb (Mouse tissue)	Cell Signaling Technology	3395	180	Rabbit
LRP6 (C5C7) Rabbit mAb (Rat tissue)	Cell Signaling Technology	2560	180	Rabbit
β-Catenin(6B3)Rabbit mAb	Cell Signaling Technology	9582	102	Rabbit
p-Smad2/3(C47E12)Rabbit mAb	Cell Signaling Technology	8828	52，60	Rabbit
Active β-Catenin(C47E12)Rabbit mAb	Cell Signaling Technology	8814	102	Rabbit
Rabbit(DA1E) mAb IgG	Cell Signaling Technology,	3900	50	Rabbit
Collagen I Rabbit pAb	Millipore	3072327	130	Rabbit
Wnt5a Rabbit pAb	GeneTex， Inc.	GTX111187	42	Rabbit
Wnt11 Rabbit pAb	GeneTex， Inc.	GTX105971	43	Rabbit
Collagen III Rabbit pAb	Proteintech	22734-1-AP	139	Rabbit
MMP2 Rabbit pAb	Proteintech	10373-2-AP	72	Rabbit
MMP9 Rabbit pAb	Proteintech	10375-2-AP	92	Rabbit
TGF-β1 Rabbit pAb	Abcam	ab92486	44	Rabbit
Alpha-SMA Rabbit mA	Abcam	ab124964	42	Rabbit
Anti-flag tag	Abcam	Ab205606	-	Rabbit
Anti-His tag	Abcam	Ab213204	-	Rabbit
Cathepsin D Antibody	Proteintech	21327-1-AP	46	Rabbit
Anti-Myc tag	Abcam	Ab32	-	Mouse
p-LRP6 Rabbit pAb	Cell Signaling Technology	2568	180	Rabbit

Table S3. Basic echocardiographic index of MCM, LRP6^{CAG}/MCM and LRP6^{CAG} male mice after tamoxifen treatment.

	MCM	LRP6^{CAG}/MCM	LRP6^{CAG}
Number	13	13	6
HR (bpm)	425.4±11.11	437.8±11.33	407.3±14.53
LVAW;d (mm)	0.77±0.02	0.80±0.03	0.76±0.02
LVPW;d (mm)	0.75±0.02	0.79±0.03	0.73±0.02
LVID;d (mm)	4.02±0.06	3.95±0.08	4.07±0.04
LVAW;s (mm)	1.17±0.03	1.18±0.06	1.12±0.06
LVPW;s (mm)	1.15±0.04	1.13±0.05	1.12±0.03
LVID;s (mm)	2.62±0.09	2.52±0.09	2.58±0.07
EF (%)	62.90±2.29	66.36±2.42	65.91±2.68
FS (%)	33.96±1.64	36.61±1.91	36.15±2.07

7-8 weeks male mice were injected tamoxifen for three days, after 1 week, parameters were analyzed. The data were expressed by mean±SEM, HR: Heart rate; LVAW;d; left ventricular diastolic anterior wall thickness; LVPW;d: left ventricular diastolic posterior wall thickness; LVAW;s: left ventricular systolic anterior wall thickness; LVPW;s: left ventricular diastolic posterior wall thickness; LVID;d: left ventricular diastolic inner dimension; LVID;s: left ventricular systolic inner dimension. EF: ejection fraction; FS: fraction shortening.

Table S4. HW/BW in MCM, LRP6^{CAG}/MCM and LRP6^{CAG} mice after tamoxifen treatment.

	MCM	LRP6^{CAG}/MCM	LRP6^{CAG}
Number	5	5	5
BW(g)	28.83±0.66	26.96±0.86	28.80±1.17
HW(mg)	137.3±4.78	131.3±7.00	137.3±6.00
HW/BW(mg/g)	4.76±0.14	4.86±0.14	4.77±0.11

7-8 weeks male mice were injected tamoxifen for three days, after 1 week, parameters were analyzed. The data were expressed by mean±SEM, BW: body weight; HW: heart

weight; HW/BW: the ratio of heart weight to body weight.

Table S5. Nano-HPLC-MS/MS analysis of the proteins interacted with LRP6 in stretched-cardiomyocytes (The unique peptide number ≥3).

	Accession	Protein	Description	#Unique peptide
1	P00507	AATM	Aspartate aminotransferase	12
2	Q8VHF5	CISY	Citrate synthase	7
3	P56574	IDHP	Isocitrate dehydrogenase [NADP]	7
4	P32551	QCR2	Cytochrome b-c1 complex subunit 2	7
5	D4AE41	RMXL1	RNA binding motif protein, X-linked-like-1	6
6	P10760	SAHH	Adenosylhomocysteinase	5
7	P15650	ACADL	Long-chain specific acyl-CoA dehydrogenase	5
8	P17764	THIL	Acetyl-CoA acetyltransferase	5
9	P15999	ATPA	ATP synthase subunit alpha	5
10	P08503	ACADM	Medium-chain specific acyl-CoA dehydrogenase	5
11	P13437	THIM	3-ketoacyl-CoA thiolase	4
12	Q9EPH2	MRP	MARCKS-related protein	4
13	P51635	AK1A1	Alcohol dehydrogenase [NADP(+)]	4
14	P12007	IVD	Isovaleryl-CoA dehydrogenase	4
15	P24268	CTSD	Cathepsin D	3
16	P60711	ACTB	Actin, cytoplasmic 1	3
17	P62738	ACTA	Actin, aortic smooth muscle	3
18	P50753	TNNT2	Troponin T, cardiac muscle	3
19	A1A5P2	RRS1	Ribosome biogenesis regulatory protein homolog	3
20	P05065	ALDOA	Fructose-bisphosphate aldolase A	3
21	P63018	HSP7C	Heat shock cognate 71 kDa protein	3
22	P16617	PGK1	Phosphoglycerate kinase 1	3

Table S6. The peptide sequences of Cathepsin D were identified by Nano-HPLC-MS/MS analysis.

Accession	Protein	Sequence
P24268	Cathepsin D	QPGVVFIAAK
		EPVSELLK
		SDLGGIKVEK

Table S7. Genotype and clinical phenotypes of Dilated cardiomyopathy (DCM) patients carrying the missense mutation of LRP6 or CTSD that is absent in controls.

Gene	LRP6	CTSD
Mutation	c.C3310T	c.G1138A
Amino acid	p.P1104S	p.G316R
Patient Number	A101	A27
Sex	Male	Male
Age	31	36
SBP	140	142
DBP	100	91
HR	70	87
EF	33%	29%
Diagnosis	DCM	DCM

LPR6:Low-Density Lipoprotein Receptor-Related Protein 6; CTSD: cathepsin D; SBP: Systolic blood pressure; DBP: Diastolic blood pressure; HR: Heart rate; EF: ejection fraction;

Supplemental figures and figure legends

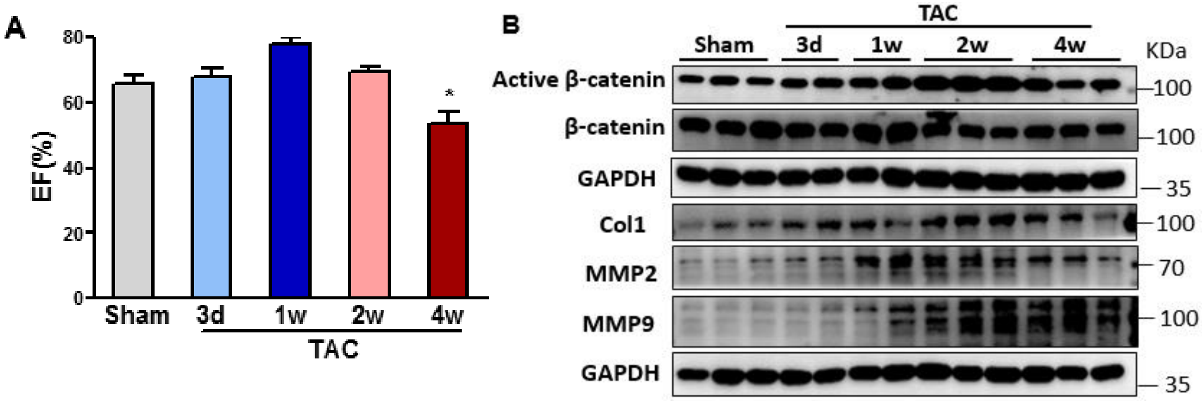

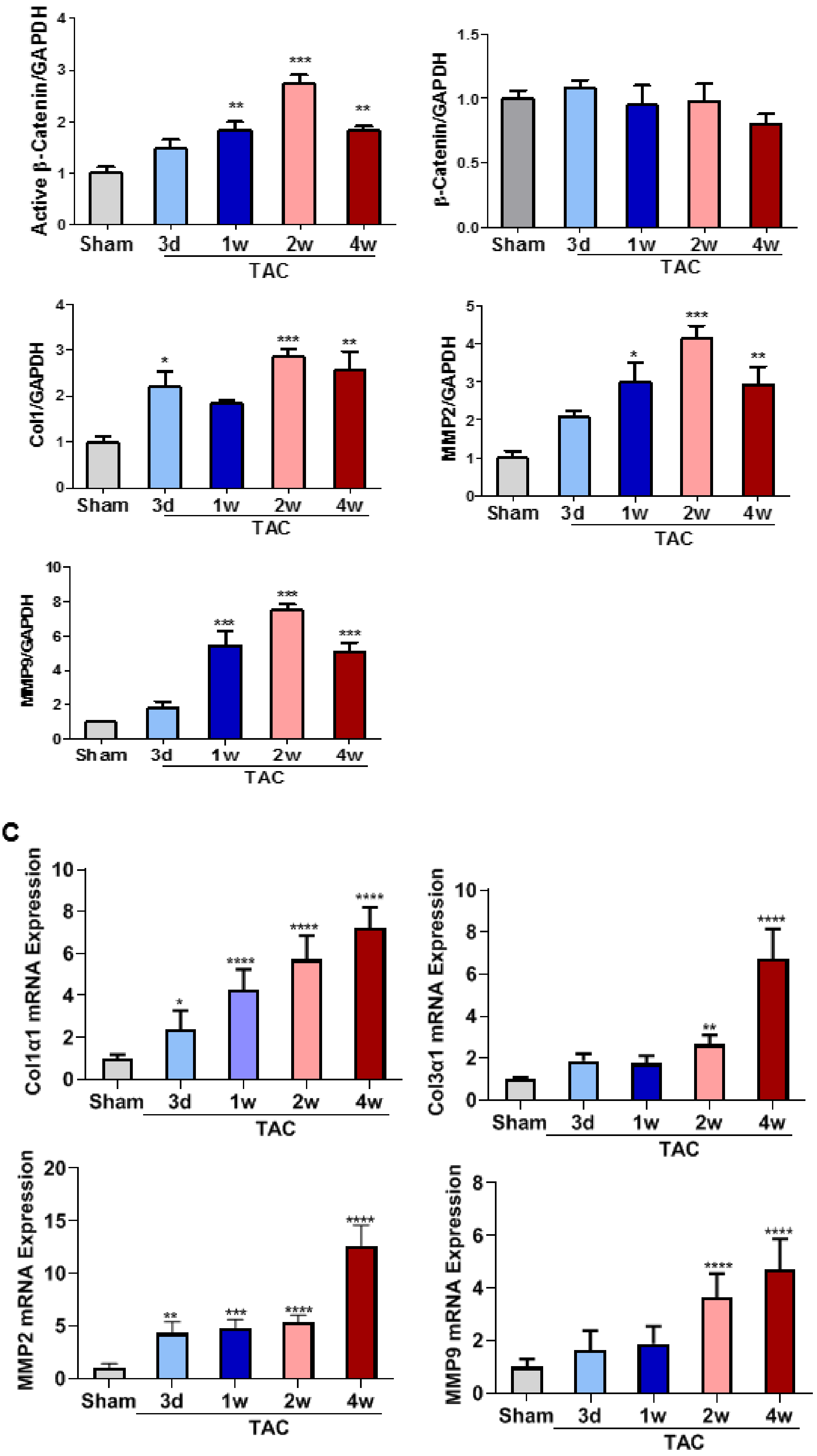

Figure S1. Cardiac function and fibrotic-related proteins or genes are evaluated in mice

at different time-points (3d, 1w, 2w and 4w) after TAC. A, Echocardiographic analysis of ejection fraction (EF). *p < 0.05 *vs* Sham group; n=6-10 mice/each group. B, Western blot analysis of active β-catenin, β-catenin, Col1, MMP2 and MMP9 expression. *p < 0.05; **p < 0.01; ***p < 0.001 vs sham group; n = 3 mice/each group. C, Real-time-PCR analysis of the mRNA level of Col1, Col3, MMP2 and MMP9. *p < 0.05; **p < 0.01; ***p < 0.001; ****p < 0.0001*vs* sham group; n = 3 mice/each group.

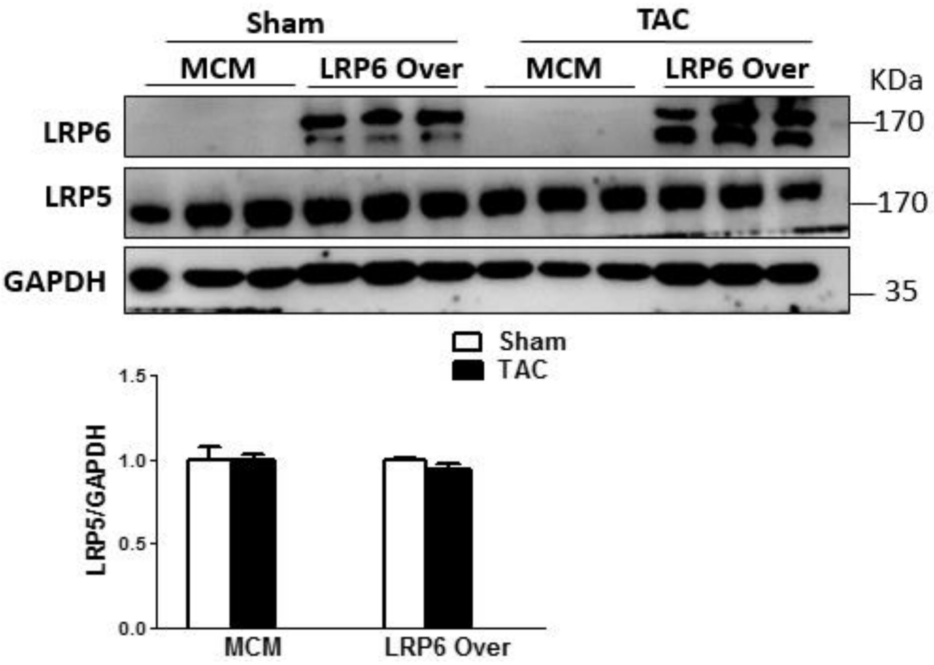

Figure S2. Western blot analysis of LRP5 expression in heart tissue from cardiac LRP6 overexpressing (LRP6 Over) or tamoxifen-injected-MCM (MCM) mice at 4 weeks after TAC or sham operation. n=3/each group.

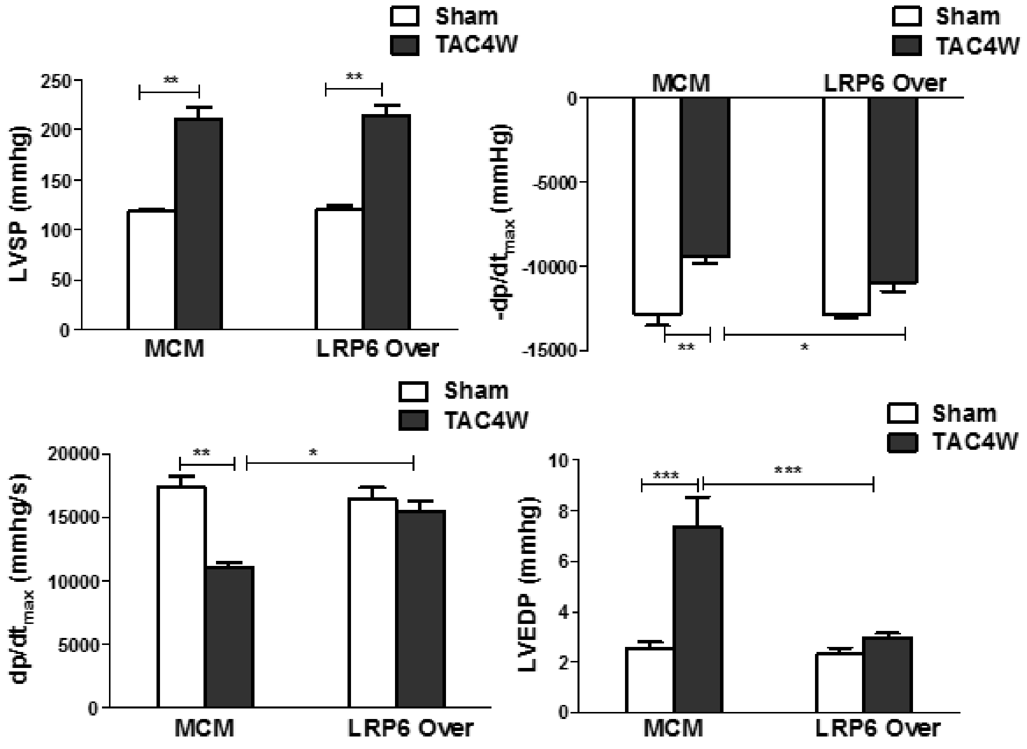

Figure S3. Hemodynamic analysis of LVSP, LVEDP, dp/dt_{max}, and $-dp/dt_{max}$ in tamoxifen-injected-LRP6^{CAG}/MCM (LRP6 Over) or –MCM (MCM) mice at 4 weeks after TAC or sham operation. LVSP: left ventricular systolic pressure; LVEDP: left ventricular end diastolic pressure; *p<0.05; **p<0.01; ***p<0.001; n=6-7/each group.

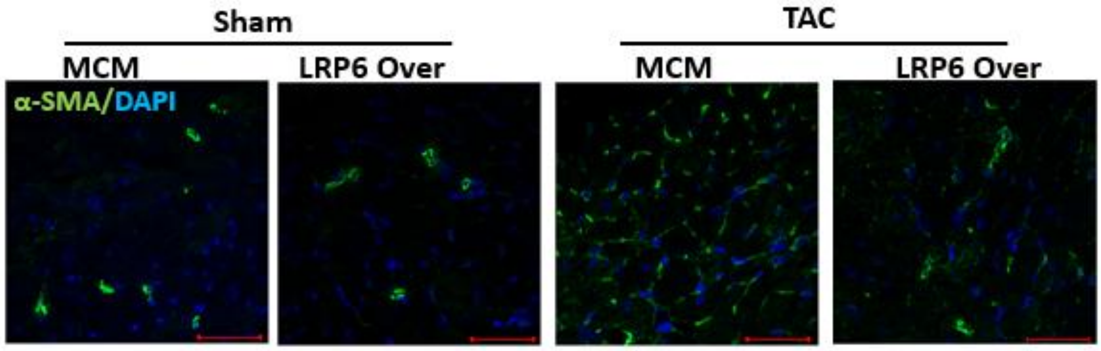

Figure S4. α-SMA immunofluorescence staining of heart tissue, Scale bars, 50um. Tamoxifen-injected-LRP6^{CAG}/MCM (LRP6 Over) or –MCM (MCM) mice were analyzed at 4 weeks after TAC or sham operation.

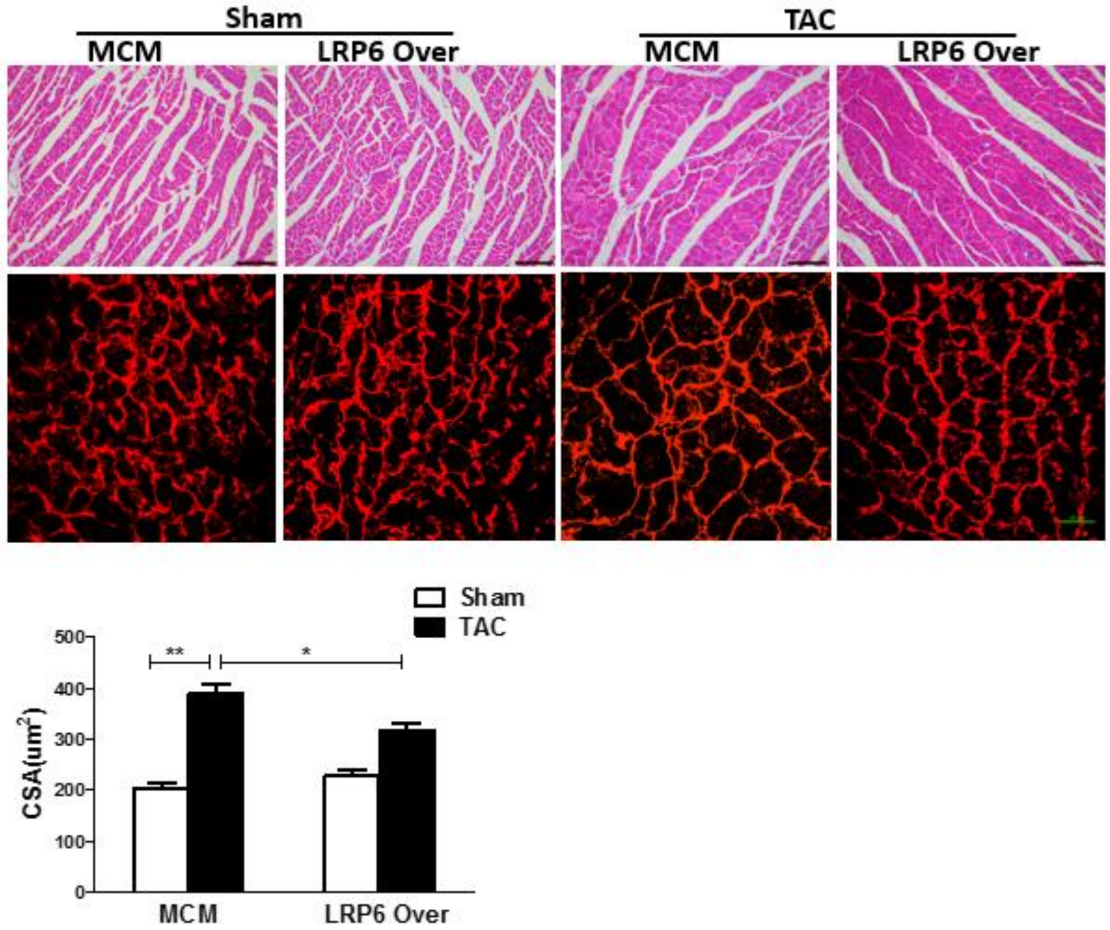

Figure S5. Cardiac specific LRP6 overexpression inhibits cardiac hypertrophy induced by pressure overload. HE and *Wheat germ agglutinin* (WGA) staining of heart tissues. Upper lane: HE staining; Scale bars,100um. Down lane: WGA staining. Scale bars, 50μm. Cross section area (CSA) of cardiomyocytes was quantitatively analyzed. *p<0.05; **p<0.01; n=7-11/each group.

A

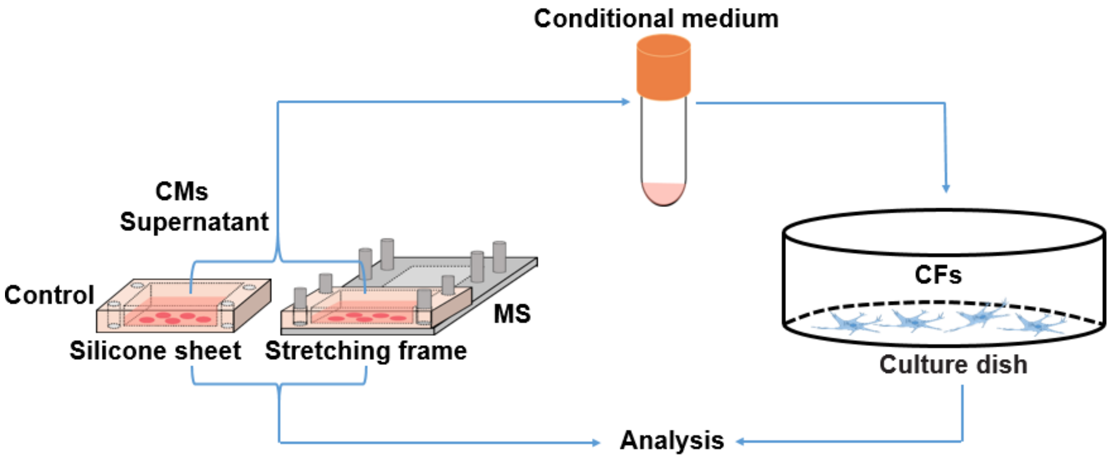

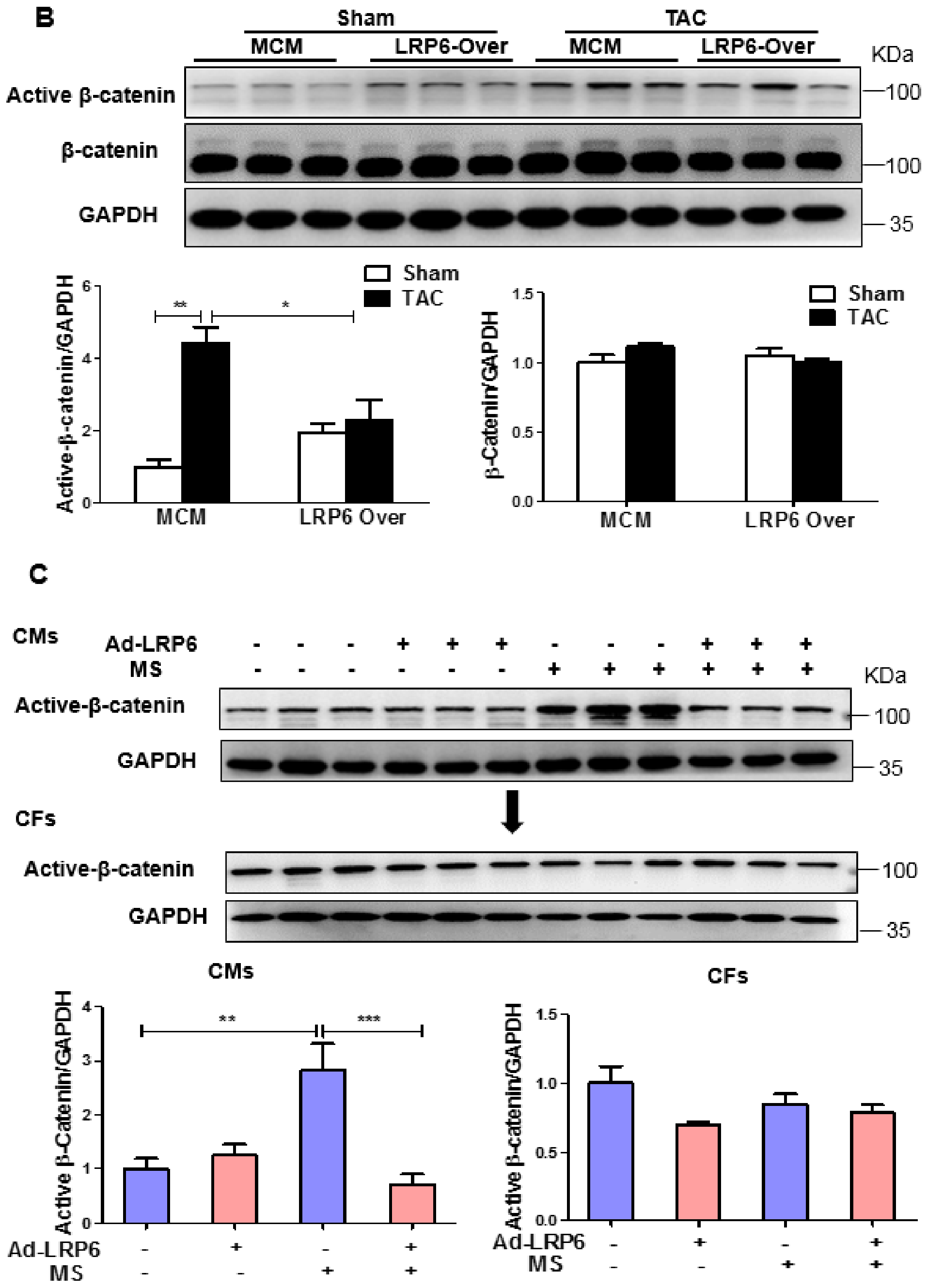

Figure S6. Cardiac specific LRP6 overexpression inhibits the activation of β-catenin. (A), Diagram of conditioned medium from stretched CMs or control CMs stimulating CFs. (B), Western blot analysis of active-β-catenin and β-catenin expression in tamoxifen-injected-LRP6^{CAG}/MCM (LRP6 Over) or MCM (MCM) mice after TAC or sham operation. *$p < 0.05$; **$p < 0.01$; n = 3/each group. (C), Western blot analysis of active-β-catenin expression in cultured CMs and CFs. Stretched (MS) or control CMs were pre-transfected with LRP6 adenovirus (Ad-LRP6) or control adenovirus (Ad-CON), and the conditioned medium from these CMs was used to stimulate CFs. **$p < 0.01$; ***$p < 0.001$; n = 6/each group.

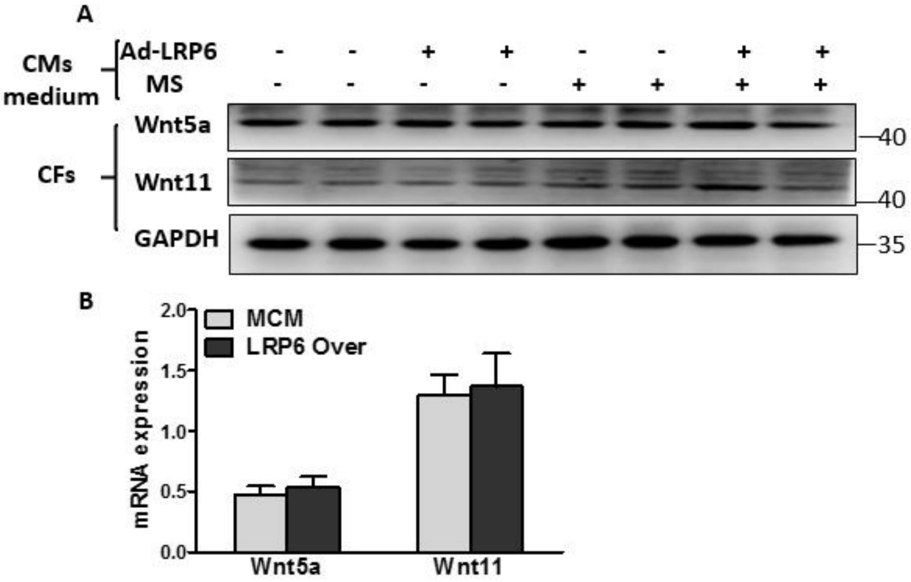

Figure S7. The effects of cardiac LRP6 overexpression on the expression of Wnt5a and Wnt11 under pressure overload. (A), The representative images of Wnt5a and Wnt11 expression (western blot analysis) in cultured CFs stimulated with the culture medium from stretched (MS) or control CMs transfected with LRP6 adenovirus (Ad-LRP6) or control adenovirus (Ad-CON). (B), Real-time PCR analysis of Wnt5a and Wnt11 mRNA expression in cardiac LRP6 overexpressing (LRP6 Over) or tamoxifen-injected-MCM (MCM) mice at 4 weeks after TAC. n=6/each group.

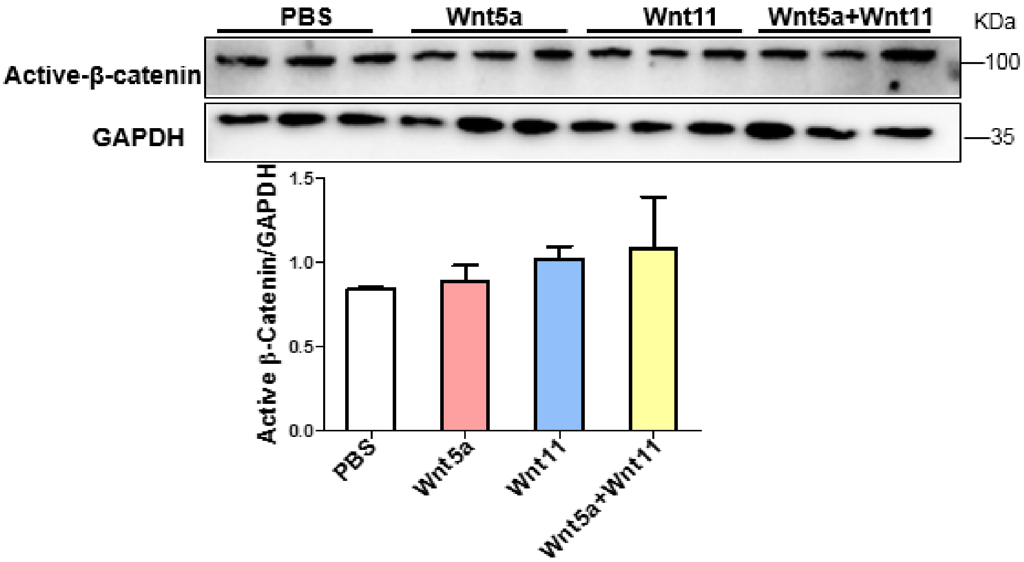

Figure S8, Western blot analysis of active β-catenin expression in CFs treated with Wnt5a, Wnt11, or Wnt5a+Wnt11, at 10 ng/mL. n=3/each group.

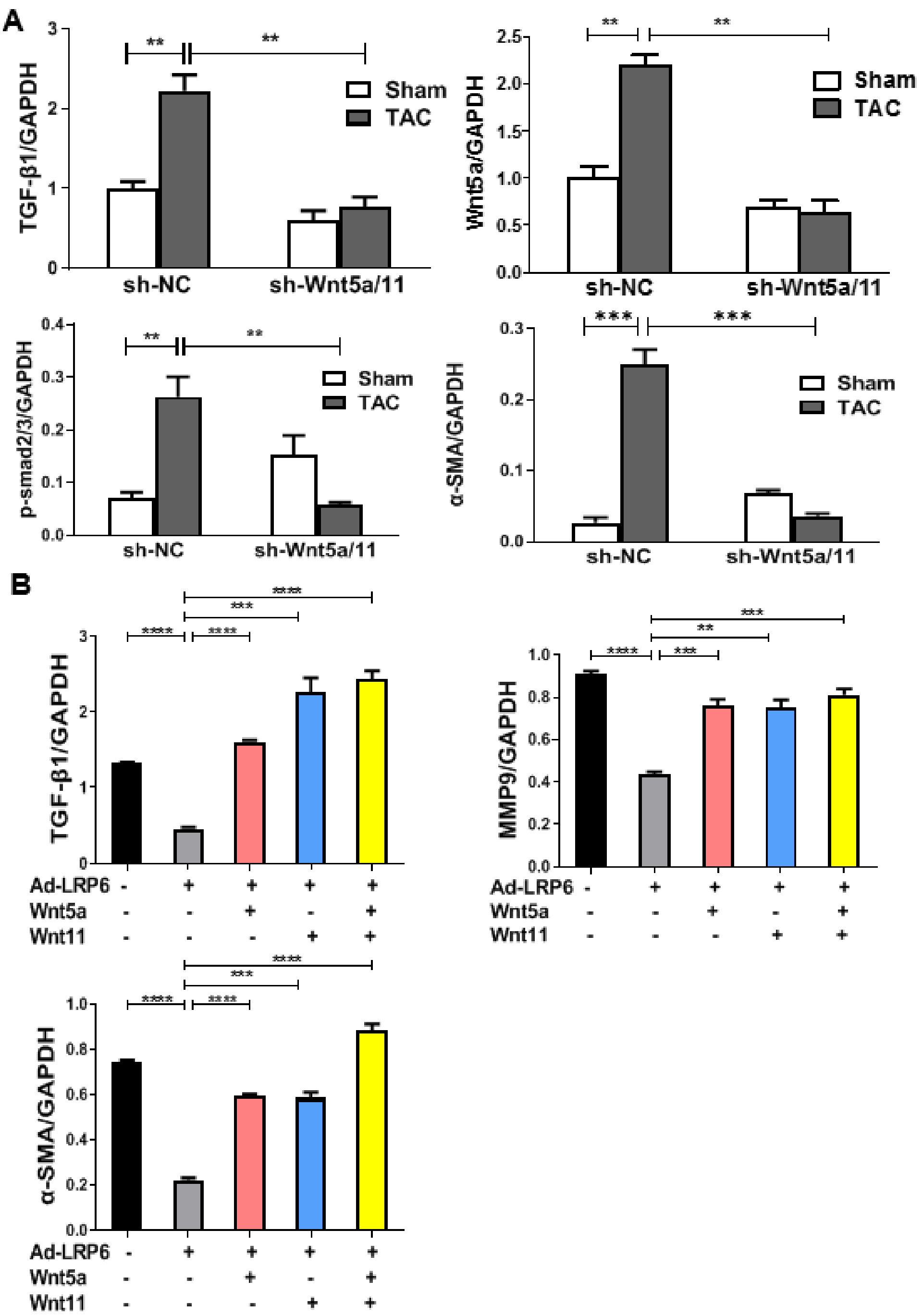

Figure S9. LRP6 overexpression inhibits cardiac fibrosis by promoting the degradation of Wnt5a and Wnt11 under pressure overload. (A), Quantitative analysis of the expression of Wnt5a, TGF-β1, α-SMA, and p-smad2/3 expression in heart tissue from TAC or sham mice pre-injected with sh-Wnt5a/Wnt11-AAV9 (sh-Wnt5a/Wnt11) or sh-scramble-AAV9 (sh-NC) by tail vein. **p < 0.01; ***p < 0.001. n=4-6 mice/each group. (B), Quantitative analysis of TGF-β1, α-SMA and MMP9 expression in CFs stimulated with the culture medium from control (Ad-CON) or LRP6 overexpressing CMs (Ad-LRP6), Wnt5a, Wnt11, Wnt5a+Wnt11 at 10 ng/mL, or PBS were supplemented with the culture medium from Ad-LRP6-CMs under MS and treated with CFs. **p < 0.01; ***p < 0.001; ****p < 0.0001. n=3/each group.

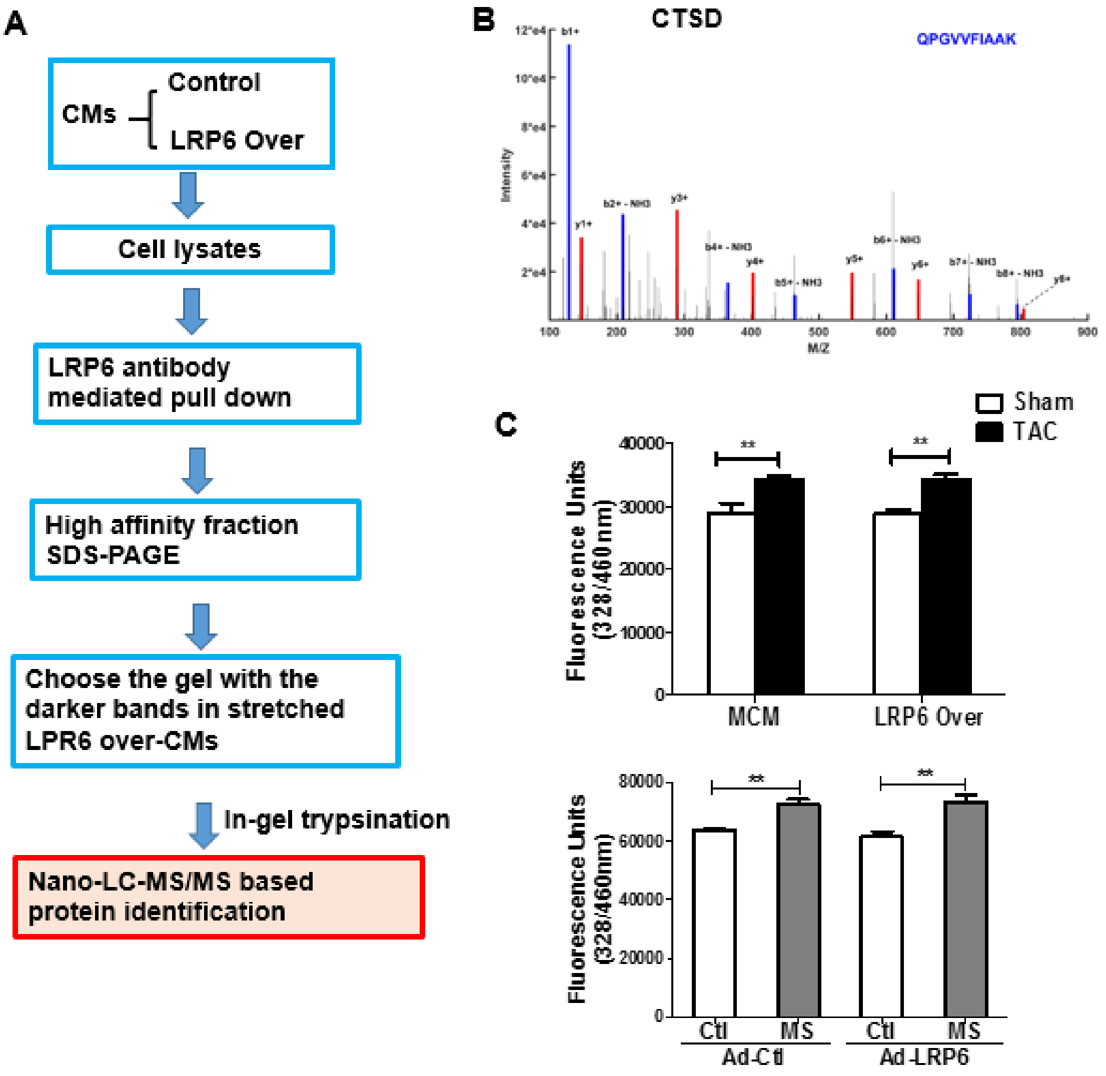

Figure S10. LRP6 overexpression enhances the interaction of LRP6 and CTSD but doesn't alter the activity of CTSD. (A), Summary of Nano-HPLC-MS/MS analysis of the proteins interacting with LRP6 in CMs under MS. (B), CTSD was identified to be interact with LRP6 by Nano-HPLC-MS/MS analysis. (C), CTSD activity was analyzed in the heart tissue from tamoxifen-injected-LRP6^{CAG}/MCM (LRP6 Over) or –MCM (MCM) mice at 4 weeks after TAC or sham operation (Upper lane) and in control (Ad-ctl) or LRP6-overexpressing CMs (Ad-LRP6) with or without MS (Ctl) (Down lane), respectively. **p < 0.01; n = 4-6/each group.

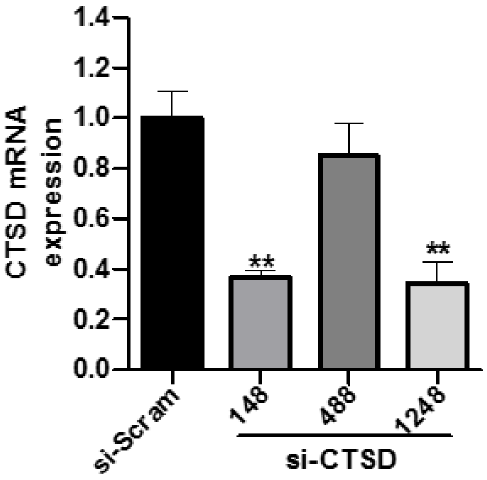

Figure S11. CTSD mRNA analysis in cardiomyocytes transfected into si-CTSD (number: 148, 488, 1248) or si-Scramble (si-Scram). **p<0.01; n=5/each group.

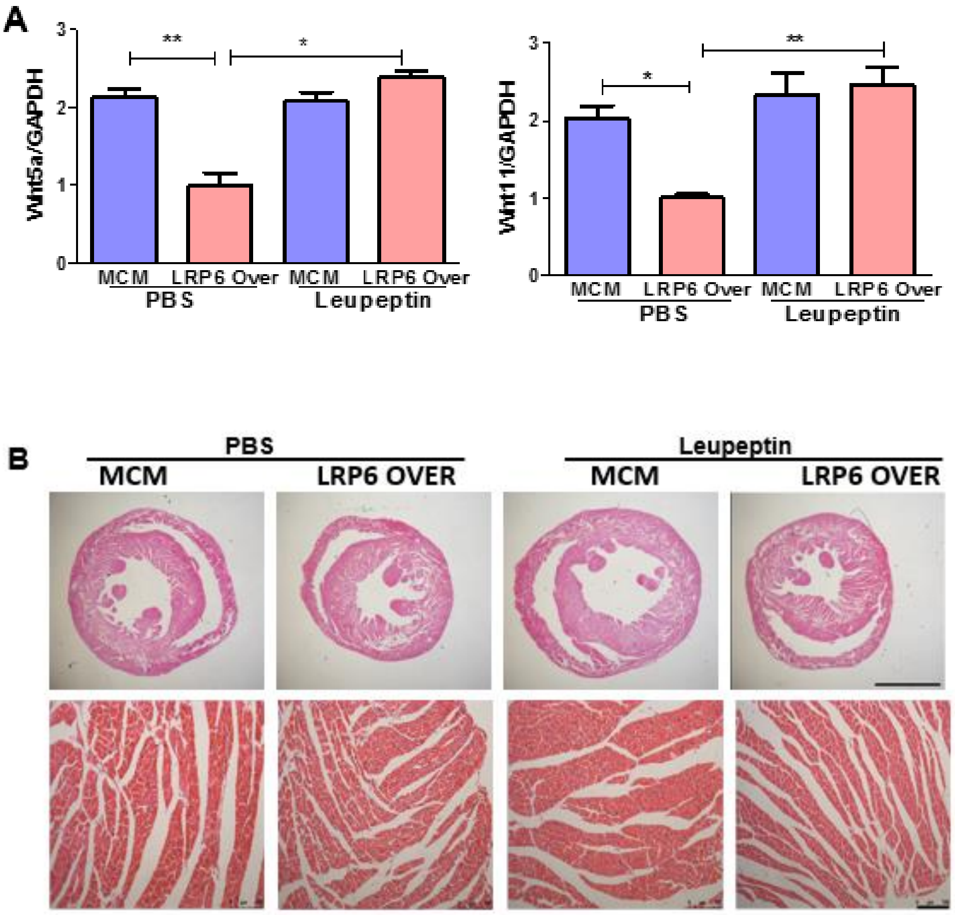

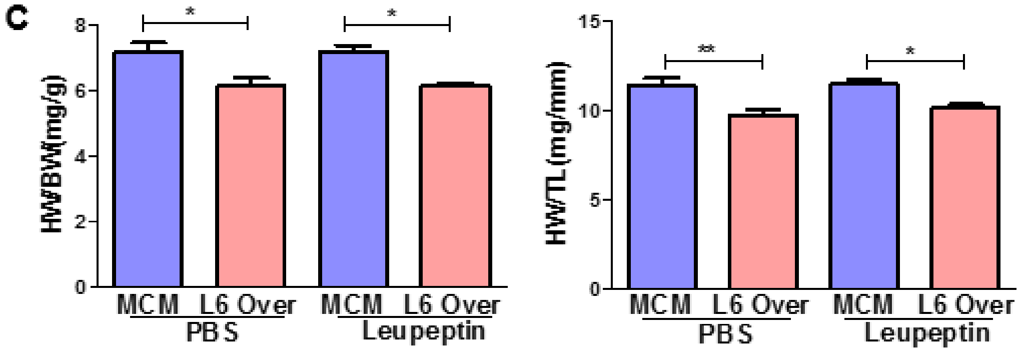

Figure S12. Protease inhibitor doesn't attenuate the inhibition of cardiac hypertrophy in LRP6 overexpressing mice after TAC. (A), Quantitative analysis of Wnt5a and Wnt11 expression. n = 3/each group. (B), HE staining of heart tissue. The upper lane: bar=2mm; The down lane: bar=100μm. (C), Quantitative analysis of heart weight/body weight ratio (HW/BW) and heart weigh/tibia length ratio (HW/TL). Protease inhibitor, Leupeptin (40mg/kg), was intraperitoneally injected to LRP6 Over and MCM mice from 2 weeks to 4 weeks (Every other day) after TAC, PBS treatment was as control. *p < 0.05; **p < 0.001; n = 7-11/each group.
